# Computational Pipeline for Accelerating the Design of Glycomimetics

**DOI:** 10.1021/acs.jcim.5c02282

**Published:** 2025-11-27

**Authors:** Yao Xiao, Alexander H. Lee, Sawsan Mahmoud, Bilqees Sameem, Daniel Wentworth, Xiaocong Wang, Grayson D. Miller, Oliver C. Grant, B. Lachele Foley, Robert J. Woods

**Affiliations:** Complex Carbohydrate Research Center, 1355 University of Georgia, 315 Riverbend Rd, Athens, Georgia 30605, United States

## Abstract

To accelerate the rational design of glycomimetic inhibitors, based on derivatization of a carbohydrate ligand, we introduce a computational pipeline that automates the creation and modeling of analogs and computes their interaction energies. Putative glycomimetics are assembled by grafting small drug-like moieties onto the native carbohydrate scaffold in the presence of the receptor protein, with the moieties chosen from a virtual library of more than 1500 molecular fragments, selected for their synthetic accessibility. The method is illustrated for the case of glycomimetics but is generalizable to any bound ligand. A genetic algorithm (GA) was developed to identify the most likely orientation of the appended moieties in the receptor binding site. For validation, curated experimental data sets were assembled from the literature, consisting of 119 glycomimetics, with reported solution binding free energies, including 46 with corresponding high-resolution crystal structures of the glycomimetic complexes. These data sets were subdivided for protocol testing and “real-world” performance validation. The GA search resulted in an average root-mean-squared deviation (RMSD) of 1.5 Å for the added moieties, compared to their crystallographic data. The GA-generated structures were then subjected to molecular dynamics (MD) simulation, and the performance was evaluated for three post-MD approaches to computing interaction energies: the scoring function from AutoDock Vina-Carb, as well as the generalized Born and Poisson–Boltzmann surface area (GBSA/PBSA) implementations within the AMBER molecular mechanical (MM) force field. For the Test data set of structures with reported energies, the highest coefficient of determination (R^2^ = 0.67) was obtained with MM-PBSA when ligand conformational entropies were included. Current limitations of the protocol and experimental data sets are discussed.

## Graphical abstract

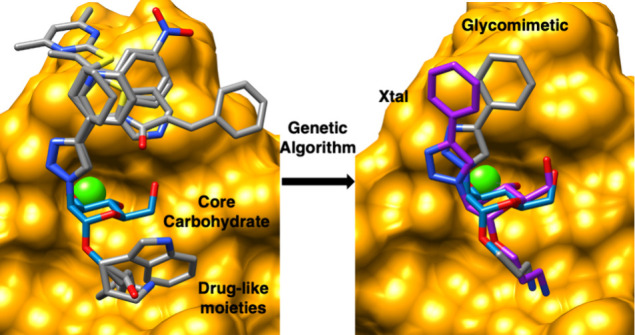

## Introduction

Endogenous protein-carbohydrate interactions play vital roles in myriad physiological events, including cell signaling,^1,2^ plant defense,^3,4^ immunity,^5^ as well as in the onset and progression of many infectious^6–10^ and noninfectious^11–15^ diseases. The inhibition or modulation of protein-carbohydrate interactions thus presents a profound therapeutic opportunity. While there are some notable carbohydrate drugs,^16^ including heparin and the synthetic heparinoid fondaparinux, the weak affinities of most carbohydrate-protein interactions (*K*_*D*_ high uM to mM),^17^ among other issues,^18^ attenuate the direct use of carbohydrates as inhibitors. One approach to overcoming this limitation is affinity amplification via avidity enhancement,^19^ which typically entails the design and synthesis of multivalent glycoconjugates, such as dendrimers^19–23^ or polymers^24,25^ that can increase the apparent affinity by over 1000-fold.^25^ However, depending on the intended application, the therapeutic potential of glycodendrimers can be complicated by cytotoxicity,^26–28^ and potentially by lower membrane permeability.^29^ Alternatively, inhibition may be achieved with traditional drug-like molecules, whose development can be facilitated through established screening and discovery methods.^30–32^ Glycomimetics provide a complementary therapeutic strategy,^33,34^ wherein the endogenous carbohydrate provides a scaffold that confers the desired receptor specificity, while affinity and drug-like properties are enhanced by appending one or more moieties at reactive centers.^35–38^ Mimetics have been generated for a broad range of biomolecules in addition to carbohydrates, including peptides^39^ and nucleic acids.^40^ As noted by Lenci et al.,^41^ decorating reactive centers in the native ligand with a variety of different appendages is the simplest way to generate structural diversity for drug discovery and lead optimization. Carbohydrates are particularly well-suited for such a strategy, as they contain a high percentage of reactive centers, most often hydroxyl groups, in a compact molecular scaffold. A number of glycomimetic compounds are currently in clinical use^16,33^ and their potential has been reviewed recently.^33,37,42^ While their promise is great, the development of mimetics remains a challenge.^33,41–43^ For example, this approach can lead to a combinatorial explosion in the number of potential analogs,^41^ beyond synthetic capacity. This problem is compounded by the notoriously laborious nature of carbohydrate synthesis.^18,44^ One innovative approach to reducing the synthetic burden leveraged click chemistry^45^ and immobilized-ligand screening to facilitate glycomimetic lead discovery.^46^ Indeed, the synthetic convenience of click chemistry has led to its regular use in the creation of libraries of putative glycomimetics.^46–48^

Computational methods can significantly assist in focusing the selection or prioritization of the choice of derivative moiety,^6,37,49^ as well as in the identification of reactive centers within the carbohydrate whose modification is likely to be the most beneficial.^50^ To promote the broader adoption of sophisticated modeling methods in glycomimetic design, we have established curated structure and affinity data sets, and used them to develop and validate a suite of freely available computational tools designed to simplify and standardize the discovery and optimization of glycomimetics.

In contrast to approaches that employ carbohydrate docking,^51–54^ the proposed workflow combines a known 3D structure for the endogenous carbohydrate bound to its receptor protein with a virtual 3D structure for the putative appendage (a.k.a. the moiety). An initial 3D structure of the glycomimetic is generated by grafting the moiety onto a reactive center in the carbohydrate scaffold in the presence of the receptor. Thus ligand docking is not performed, although a docking scoring function is used to evaluate the preferred conformation of the derivative moiety. This preferred conformation is determined in the presence of the receptor using a customized genetic search algorithm (GA). The moieties are stored in separate structure files, comprising the virtual moiety library (currently more than 1500 members), designed to facilitate their transferability to any reactive center in a carbohydrate ligand. Moieties were selected on the basis of their suitability for synthetic coupling to hydroxyl or amino groups in a carbohydrate. The accuracy of the GA search protocol was established by comparison against a curated set of 46 glycomimetic–protein crystal structures. Binding energies were computed from MD simulations of the glycomimetic complexes using a version of the AutoDock VINA scoring function^55^ optimized for carbohydrates (Vina-Carb, VC^53^), as well as by computing the molecular mechanics (MM) interaction energies with desolvation energies provided by the Poisson–Boltzmann or generalized Born surface area (MM-PBSA/MM-GBSA) approximations,^56^ augmented by ligand conformational entropies. The validated protocols were then applied to predict the interaction energies for a set of 73 glycomimetics with reported solution affinities, employing the 3D structures of the endogenous carbohydrate-receptor complexes. A total of 141 moieties were extracted from the curated structure data set, including 61 created using click chemistry, and included in the virtual moiety library. When applied to a carbohydrate with multiple reactive centers, the present library would rapidly lead to a synthetically intractable number of potential analogs, necessitating the application of a computationally guided filtering strategy, such as reported herein.

## Methods

### Curated Glycomimetic-Receptor Data Sets

A data set of protein-glycomimetic structures was created and employed for method development (the “Test” data set). Acceptable 3D structures were required to satisfy the following conditions: (1) a resolution of less than 2.5 Å, an average B-factor for the protein atoms of less than 75 Å^2^ and an average B-factor for the ligand atoms of less than 100 Å^2^ (Table S1). In addition, a data set of binding free energies for glycomimetics was assembled (Table S2). To reduce variations among reported experimental affinities, only data from solution binding assays, such as isothermal titration calorimetry (ITC), fluorescence polarization (FP), or microscale thermophoresis (MST) were included. Data from affinity methods that involved immobilization of the analyte, such as surface plasmon resonance (SPR) and biolayer interferometry (BLI) were excluded. While such assays can be performed under conditions that lead to binding constants (*K*_D_) that are equivalent to solution values,^57,58^ in general these methods only measure an apparent *K*_D_, which can differ by as much as 1000-fold from the true solution values.^59,60^

A complementary data set of glycomimetics was assembled with reported solution binding free energies, but lacking crystal structures of the complexes (the “Application” data set). In addition, for inclusion in the Application data set, crystal structures meeting the Test data set criteria must have been reported for the endogenous carbohydrate bound to its receptor.

### Genetic Algorithm

A C++ program (“GM”) was developed to perform virtual screening using a custom genetic algorithm (GA, see Supplementary Data for details) and to prepare the files necessary for subsequent MD simulations in AMBER (Figure S1). The C++ programs developed in this work take advantage of the GLYCAM Molecular Modeling Library (GMML), a C++ functional library developed in the Woods group and distributed as a GIT repository (https://github.com/GLYCAM-Web/gmml2). The GM program requires three input files: the protein-carbohydrate cocomplex (PDBQT format), a virtual library of 3D structures of derivative moieties (PDBQT format), and a file that specifies the reactive center (derivatization position) in the carbohydrate (See Supplementary Data for examples). The GA implementation of the VC scoring function employs AutoDock atom types, and was written to read an AutoDock PDBQT file as input. Although the VC scoring function was developed to correctly account for the *exo*-anomeric effect^61,62^ between monosaccharide residues, via inter-residue CHI energy functions,^63^ the conformational preferences of the aglycone were not similarly defined. Hence, the CHI energy terms were adapted to include the aglycone.

### Moiety Grafting

For aligning the moiety to the carbohydrate, and for partial charge calculations, a temporary ethyl group was introduced to the attachment atom of each moiety (R), forming R-CH2CH3, with the inner CH2 group mimicking the side-group hydroxyl, and the terminal CH3 group mimicking the adjacent ring carbon. For aligning the R group to the carbohydrate, the terminal CH3 group was superimposed on the ring adjacent carbon, followed by the rotation of the side group hydroxyl-ring carbon/CH3–CH2 angle to 0°. This operation resulted in a reasonable initial alignment of the moiety relative to the carbohydrate ring. The ethyl group was subsequently removed from the system, resulting in the proposed glycomimetic analog (R, side group hydroxyl-ring carbon), and the partial charges adjusted as described.

Prior to GA screening, each moiety in the selected library is sequentially grafted onto the specified reactive center in the monosaccharide. For each mimetic, the optimal binding pose of the moiety is determined with the GA, as outlined in Figure S1, employing the VC scoring function. Upon optimization, structure files (PDB) are output for each glycomimetic-protein complex. Four E-selectin-glycomimetics (T53, T56, T57, and T58) were excluded from GA screening, because the oligosaccharide ligands in the glycomimetics contained non-natural monosaccharide residues that were not present in the GLYCAM carbohydrate library. For these four systems, their MD simulations were started directly from the crystal structure.

### PDB2GLYCAM

A C++ program termed “PDB2GLYCAM” (Figure S2) was created to facilitate the mapping of atomic properties, assigned in the PDBQT file, to those recognizable by the AMBER/GLYCAM force fields. In addition, PDB2GLYCAM identifies each monosaccharide in the PDBQT file (created from the initial PDB structure) based on the ring size, the carbon atom configurations, and the composition of the exocyclic moieties. This approach was adopted to assist in subdividing an oligosaccharide ligand into its constituent monosaccharides, and to avoid any carbohydrate nomenclature errors that may have been present in the crystallographic data.^64–66^ Once parsed into constituent monosaccharides, the corresponding monosaccharide templates in the GLYCAM06 force field are identified and the oligosaccharide is converted into a graph structure, with nodes representing individual atoms and edges representing interatomic bonds. Subgraph isomorphism matching analysis is then performed between the carbohydrate structure identified in the PDBQT file and its counterpart in the GLYCAM06 force field template. Upon program termination, the GLYCAM06 atom types and atomic partial charges for the carbohydrate atoms in the glycomimetic are reported in the PDB2GLYCAM log file. If present, moiety atoms are also listed in the output and are marked as noncarbohydrate.

### Restrained Electrostatic Potential (RESP) Charges

Atomic coordinates of each glycomimetic (generated by the GM protocol) were optimized with the Berny geometry optimization algorithm using GEDIIS at the HF/6-31+G* level of theory using the Gaussian16 ^67^ software package. Electrostatic potential (ESP) calculations were performed for the optimized structure using the CHelpG scheme.^68^ Restrained ESP (RESP) partial atomic charges were computed for each atom by fitting the classical Coulomb model to the QM ESPs, with a charge restraint weight of 0.0001, following established RESP protocols.^69^ The HF/6-31+G* level of theory was chosen in line with the standard protocol for charge derivation in the GLYCAM force field.

### Virtual Moiety Library

Moieties were selected on the basis of their synthetic accessibility for reactions involving hydroxyl or amino groups in a carbohydrate, and in addition by the commercial availability of precursor reagents, such as aldehydes (*n* = 451), acyl halides (*n* = 177), alkyl halides (*n* = 144), sulfonyl halides (*n* = 101), and alcohols (*n* = 294). Additional moieties were added for each of the glycomimetics in the Test and Application data sets, as well by extraction from PubChem (https://pubchem.ncbi.nlm.nih.gov/), and from consultation with synthetic chemists, resulting in a total of 1543 unique moieties.

3D structures of the moieties were generated by conversion of their Simplified Molecular Input Line Entry System (SMILES)^70^ string into Cartesian coordinates (PDB format) using the code available within the RDKit (https://www.rdkit.org/) modules, followed by energy minimization using the L-BFGS-B algorithm as provided by SciPy (https://scipy.org/). SMILES strings were either exported from PubChem or from ChemDraw. AutodockTools^71–74^ was used to convert PDB files into PDBQT format.

### GM2MD

A C++ program termed “GM2MD” (Figure S2) was developed to convert the PDBQT file into a format (OFF file) suitable for use in simulations with the AMBER software suite.^75^ The GM2MD program requires four input files: the ligand binding pose from GA (PDB format), the PDB2GLYCAM log file (plain text), RESP charges for each atom (plain text), and the GAFF^76^ atom types of each moiety atom (MOL2). GAFF atom types were assigned to each moiety atom with the Antechamber^77^ program (MOL2 format). Atomic charges for the moieties were assigned from the glycomimetic RESP calculations, while those for the carbohydrates were taken from the GLYCAM force field.

The GLYCAM charges were derived using a similar RESP protocol,^78^ but were also conformationally averaged.^79^ This approach enabled the moieties to be readily employed with the established GLYCAM monosaccharide templates. Following the general approach to charge transferability in GLYCAM,^80^ any nonintegral net charge, typically smaller than 0.1 au, that arose from grafting a library moiety into a GLYCAM monosaccharide, was corrected by evenly distributing the charge difference between the carbohydrate linkage atom (the reactive center) and the linkage atom in the moiety. Upon program termination, the fully parametrized glycomimetic ligand is output in OFF file format.

### System Preparation, Energy Minimization, and MD Simulation

In the case of multimeric receptors, a single protein chain was selected for analysis, with the only exception being in the case of cholera CT system, for which analyses were performed on both the monomeric and pentameric structures. Any structurally relevant ions located in the binding sites were retained; irrelevant ions and cocrystallization agents were removed. Hydrogen atoms were added using Chimera,^81^ which was also used for hydrogen bond analysis. Any waters that were predicted to simultaneously display hydrogen bonds to both the ligand and the receptor were categorized as bridging waters and retained during GA screening and subsequent MD simulation. Amino groups and carboxylic acids in the ligand or protein were treated as ionized. The tLEAP program in AMBERTOOLS^82^ was used to read in the protein–glycomimetic OFF files generated by GM2MD, to add counterions as necessary to ensure charge neutrality, to solvate the complex with explicit TIP3P waters, and to output the coordinate (CRD) and topology (TOP) files necessary for energy minimization and MD simulation with AMBER.

Each system was solvated within a truncated octahedron box, with a minimum distance of 10 Å between the solute and the periodic boundary. Energy minimizations and MD simulations were performed with AMBER14.^83^ The numbers of explicit water molecules for each system are provided in Table S1. A nine-step minimization/equilibration protocol recommended by Roe and Brooks^84^ for preparing explicitly solvated systems for stable MD simulations was adopted with minor modifications. Specifically, an additional restraint, with a weak force constant of 10 kcal/mol/Å^2^, was applied to all the Cα atoms in the proteins, unless otherwise specified. A production MD run of 100 ns was performed for each glycomimetic with this restraint. Hydrogen atoms were excluded from all RMSD calculations in this work.

### Ligand Binding Energies

Average binding energies were computed for 10,000 frames selected evenly from the 100 ns production trajectory by rescoring with the VC scoring function, using a C++ program (ScoreTraj), as well as using the molecular mechanics (MM) energies augmented with desolvation energies either from the generalized Born model (GBSA) or the Poisson–Boltzmann surface area methods (PBSA) as implemented in the Amber software suite. For the GBSA calculations, the GB_1_^OBC^ parametrization^85,86^ was employed (igb = 2), as it was previously reported to perform well on carbohydrate–protein complexes.^87^ The *mbondi2* radii set was employed as recommended^86^ for use with the GB_1_^OBC^ model. For the PBSA calculations, the level set function was employed to compute the dielectric interface^88^ (ipb = 2). Water molecules were removed from the trajectories prior to performing the MM-GB/PBSA analyses.

## Results

### GA Screening

As part of the development of the GA screening approach, the predicted conformations for the glycomimetics in the Test data set (Table S1) were compared to the corresponding experimental 3D structures. During the initial evaluation of the GA-generated ligand poses, it was observed that for the LecA system, the RMSD values for the glycomimetic moieties, relative to their experimental structures, were as high as 12 Å, with an average value of 5.5 ± 3.9 Å (Figure 1, yellow).

**Figure 1:**
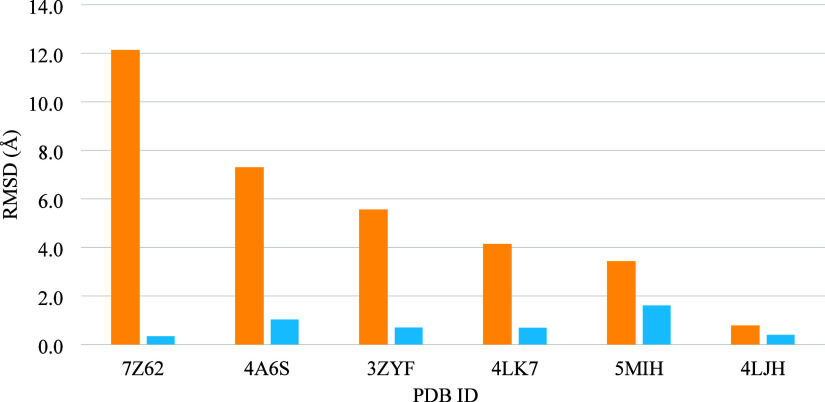
RMSD values for the glycomimetic moieties from GA screening of LecA-glycomimetic complexes with (blue) and without (yellow) inclusion of torsion terms for the *exo*-anomeric effect in the ligand.

An examination of the LecA glycomimetics (T15, 17, 24, 29, 45, 48) indicated that they all contained β-galactose scaffolds with aromatic aglycones; that is, the aromatic moieties were connected at the anomeric carbon atom in the carbohydrate. The rotamer preferences of a carbohydrate-aglycone linkage (the φ-angle) are governed by the *exo*-anomeric effect. This conformational preference generally leads to the aglycon adopting a *gauche* orientation with respect to the monosaccharide ring oxygen atom, as observed in the LecA crystal structures; whereas the moiety poses from GA screening all displayed a *trans* value for this angle (Figure 2).

**Figure 2:**
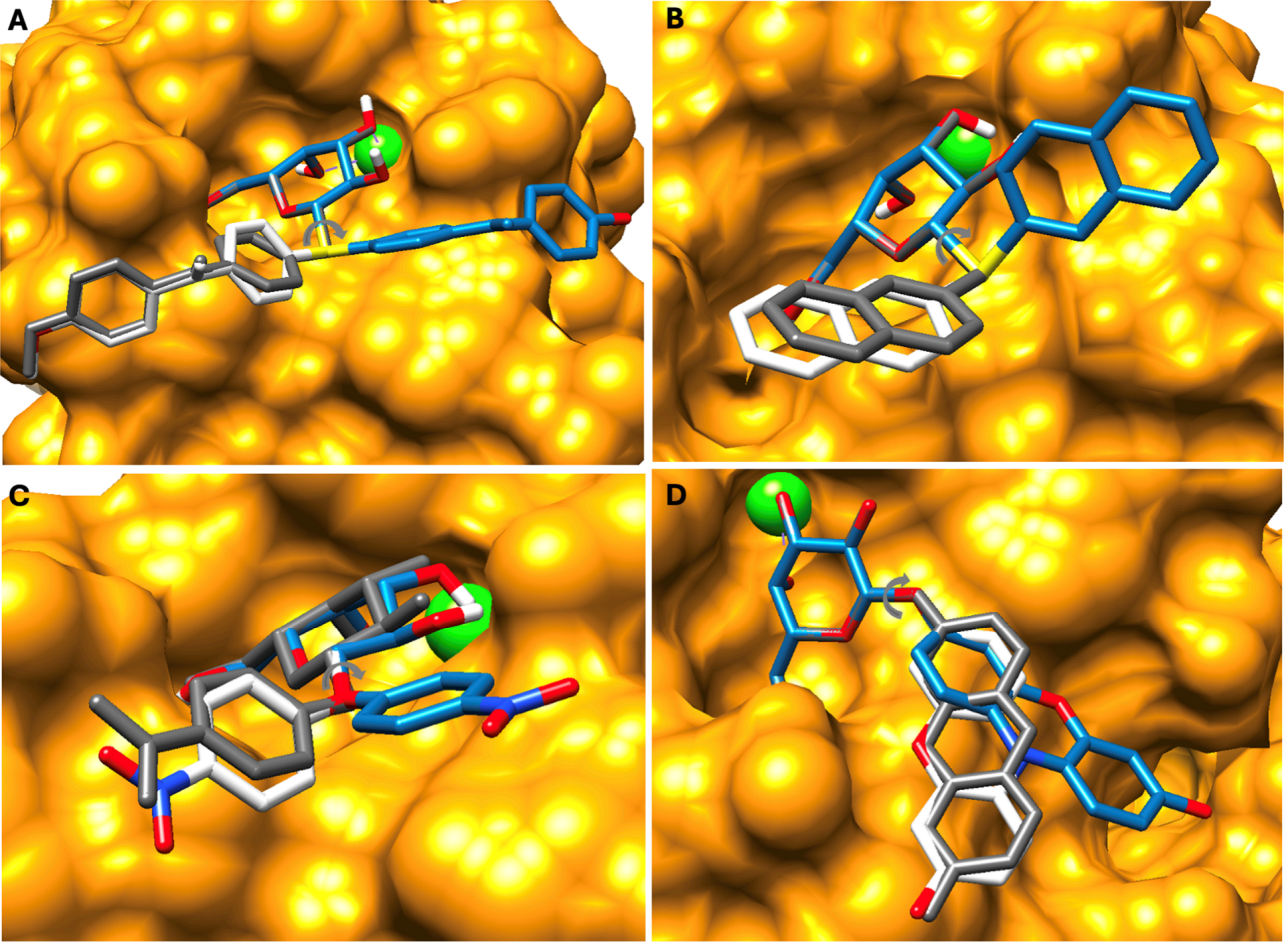
GA screening results for LecA systems with (white) and without (blue) energy terms for the *exo*-anomeric effect compared to the experimental structures (orange for protein and gray for ligand). (A) PDB ID: 7Z62. (B) PDB ID: 4A6S. (C) PDB ID: 3ZYF. (D) PDB ID: 4LK7. The anomeric φ torsion angle is indicated with a gray arrow.

After adaptation of the VC function to include CHI energy terms for the aglycone, the φ-angles were subsequently predicted to prefer the expected *gauche* conformation, leading to a dramatic reduction in the LecA moiety RMSD values (average RMSD = 0.8 ± 0.5 Å, Figure 1, blue). The CHI energy terms were therefore employed during GA screening for all glycomimetic systems.

The average RMSD for the GA-predicted moiety conformations, after inclusion of the *exo*-anomeric correction, was 1.5 ± 1.2 Å, with 67% of the predicted ligand poses being within 2 Å of the experimental position, and 87% within 3 Å (Table S3 and Figure S3). To remove bias, the crystallographic moiety conformations were randomized prior to beginning GA screening. Subsequently, the GA-generated and experimental complexes were subjected to MD simulation prior to binding energy analysis.

### MD Simulation

Although a rigorous protocol, designed to enhance the stability of MD simulations, was implemented for all systems,^84^ it was observed that not all ligands remained stably bound during the simulations. Upon closer examination, the issues were principally associated with the cholera toxin (CT) system. Although CT naturally exists as a pentamer, only a monomer was employed during the initial MD simulations, which raised the possibility that incomplete quaternary structure might be responsible for compromising ligand stability, potentially by increasing the flexibility of the protein domain. To assess the importance of this issue, MD simulations of the four CT–glycomimetic systems (T23, T40, T41, and T42) were also performed for the pentameric protein structures (Table 1). The inclusion of the full protein structure reduced the ligand RMSD from greater than 7 Å to just over 2 Å (Table 1).

**Table 1:** Average Ligand RMSD (Å) Values for LecA and CT-Glycomimetic Complexes with (Cα (+)) and without (Cα (-)) Restraints on the Protein Cα Atoms

	carbohydrate		moiety		protein	
	Cα (-)	Cα (+)	Cα (-)	Cα (+)	Cα (-)	Cα (+)
average RMSD values for LecA complexes						
GA	1.1 ± 1.2	0.5 ± 0.2	3.3 ± 1.5	2.8 ± 1.4	1.4 ± 0.1	1.0 ± 0.04
X-tal	0.7 ± 0.2	0.5 ± 0.2	3.1 ± 1.3	2.0 ± 1.4	1.5 ± 0.1	1.0 ± 0.03
average RMSD values for CT complexes						
monomeric (GA)	7.2 ± 7.2	0.5 ± 0.10	10.4 ± 5.7	3.2 ± 1.0	2.2 ± 0.1	1.2 ± 0.10
monomeric (X-tal)	7.7 ± 6.8	0.5 ± 0.03	9.6 ± 6.4	3.2 ± 1.7	2.5 ± 0.1	1.2 ± 0.02
pentameric (GA)	4.4 ± 5.3	0.5 ± 0.10	6.1 ± 6.0	3.5 ± 1.8	1.6 ± 0.1	1.0 ± 0.04
pentameric (X-tal)	2.2 ± 2.0	0.5 ± 0.10	4.0 ± 3.4	1.5 ± 0.9	1.3 ± 0.2	1.0 ± 0.03

To test whether the ligand instability arose from excessive fluctuations in the monomeric protein fold, each of the four monomeric CT systems was also subjected to MD simulations with a weak Cartesian restraint applied to the protein backbone Cα atoms. Under these conditions, the ligands in the monomeric CT–glycomimetic complexes remained as stable as those in simulations of the pentameric complexes (Table 1 and Figure S4).

Interestingly, MD simulations with the monomeric LecA protein, which naturally exists as a tetramer, indicated only modest degradation of the protein structure (RMSD approximately 1.4 Å), even without Cα restraints. In addition, the ligand remained stably bound, regardless of the presence or absence of Cα restraints (Table 1). The results from the initial MD studies of the CT and LecA systems (10 complexes in total) indicated that the application of Cα restraints generally stabilized ligand binding and could compensate for a lack of complete protein quaternary structure in the MD simulation. As monomeric systems are faster to simulate than larger multimers, and their use eliminated the need for system-specific MD simulation protocols, all subsequent simulations were performed on monomeric receptors with Cα restraints.

Once the *exo*-anomeric effect and protein stability issues had been accounted for, each of the GA-generated poses for the 58 systems in the Test data set was solvated, energy minimized, and subjected to 100 ns of MD simulation. The average post-MD RMSD of the carbohydrate cores and the derivative moieties were 0.7 ± 0.4 Å and 2.5 ± 1.2 Å respectively, which were indistinguishable from the values obtained when the MD simulations were initiated from the experimental crystal structures (Table S4 and Figure S5). Post-MD, approximately 44% (*n* = 24) of the moieties remained within 2 Å of the experimental ligand position, and 76% (*n* = 41) within 3 Å. The increased RMSD values from MD simulation, compared to the results from GA screening or crystallography, are consistent with increased atomic fluctuations present in solution at room temperature.

### Interaction Energies

Having established the accuracy of the GA/MD protocol for the Test data set of protein–glycomimetic structures, these systems were then employed in the calculation of ligand interaction energies. The theoretical energies were assessed by comparison against corresponding experimentally determined binding free energies (Table S2). While many computational methods exist for predicting interaction energies, we examined three representative methods, namely, one that employed the empirical VC scoring function,^53^ and two that employed the AMBER/GLYCAM molecular mechanical energies, augmented with desolvation free energies via the generalized Born or Poisson–Boltzmann surface area approximations (MM-GBSA and MM-PBSA, respectively). Entropy changes associated with ligand binding can be large,^89,90^ yet they are often excluded from MM-GBSA/PBSA analyses, due primarily to the fact that they are notoriously slow to converge on MD timescales.^91–93^ An alternative to computing complete binding entropies is to consider only the change in ligand conformational entropy that arises from the decrease in conformational freedom upon binding. This entropy penalty has been estimated to be approximately 0.5 kcal/mol per restricted rotatable bond;^94^ with a proposed range of 0.3–1.0 kcal/mol, depending on the calculation method.^90,95^ Thus a simple rotamer-based estimate was adopted to account in part for ligand conformational entropy changes. The impact of a range of per-bond entropy penalties (0.0–1.0 kcal/mol) was examined, and a value of 0.6 kcal/mol was adopted (Table S5). Under all conditions evaluated, inclusion of conformational entropies improved correlations (R^2^) with the experimental binding free energies, in some cases quite significantly, and in no cases degraded the performance. For the most accurate post-MD data set, comprising 28 complexes with moiety RMSD values below 2.0 Å (Table 2), an R^2^ value of 0.54 was achieved with the VC scoring function with the inclusion of conformational entropy, compared to an R^2^ value of 0.40 without an entropic correction. Both MM-GBSA and MM-PBSA outperformed VC, with R^2^ values of 0.53/0.49 and 0.67/0.62, respectively, with/without inclusion of conformational entropy (Figure 3 and Table 2).

**Figure 3:**
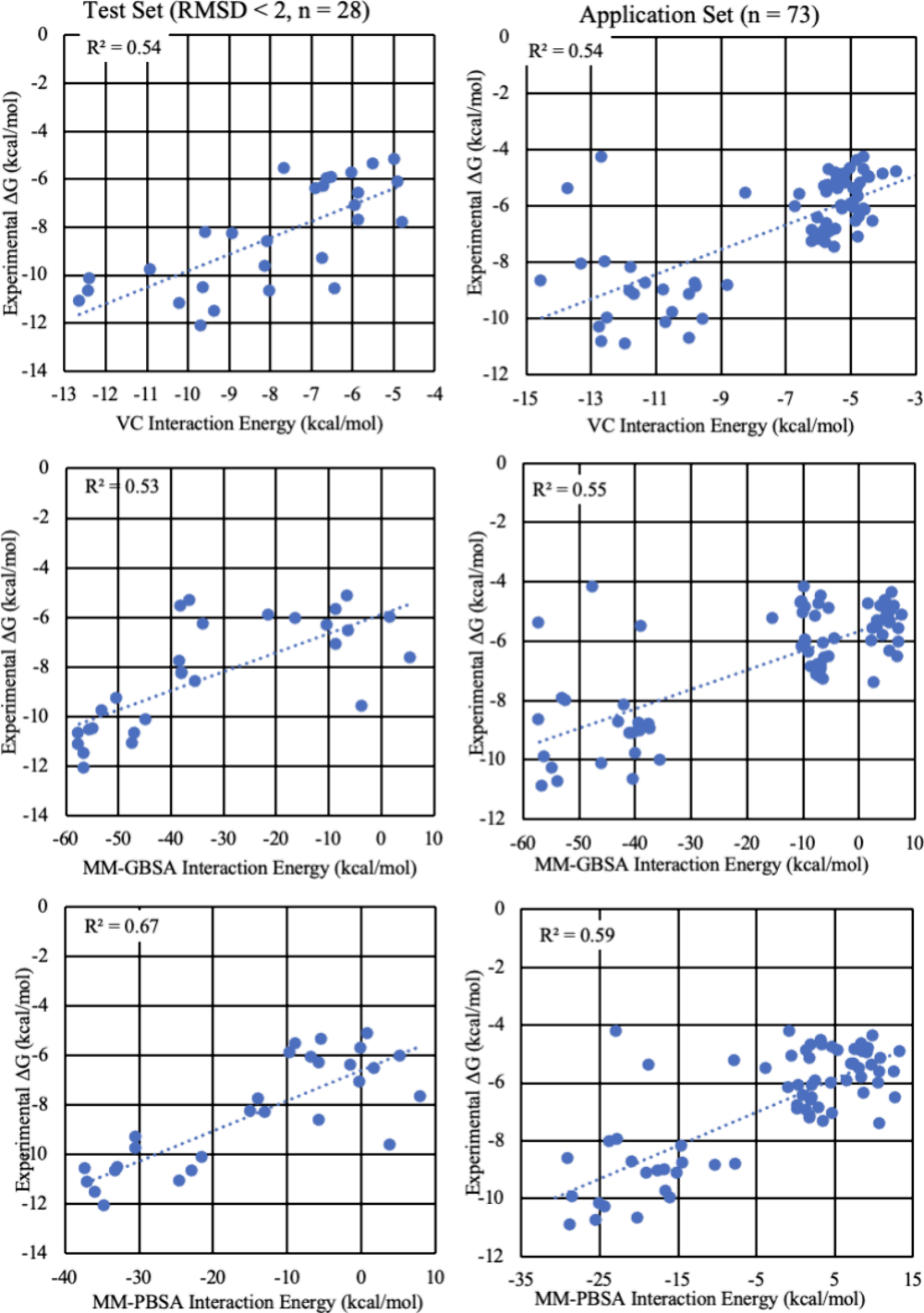
Average theoretical interaction energies from MD data versus experimental values. Left column, data from the Test data set for moieties with RMSD values < 2 Å (*n* = 28); right column, data from the Application data set (*n* = 73). Top, energies computed with the VC scoring function; middle, computed values from MM-GBSA; bottom, computed values from MM-PBSA. All theoretical values include conformational entropy.

**Table 2:** Correlation Coefficients (R^2^) for the Test Dataset from VC, MM-GBSA, and MM-PBSA Analyses Post-MD with and without Ligand Conformational Entropies

method	including entropy	not including entropy
**MM-PBSA** ^a^		
no receptors containing Ca^2+^	0.70	0.64
all receptors	0.67	0.62
**MM-GBSA** ^a^		
no receptors containing Ca^2+^	0.65	0.56
all receptors	0.53	0.49
**VC** ^a^		
no receptors containing Ca^2+^	0.28	0.15
all receptors	0.54	0.40

a Moiety RMSD < 2 Å.

The improved performance of rescoring with MM-GBSA and MM-PBSA over values from scoring functions (such as, AD 4.2.3,^71–74^ SMINA,^96^ and LibDock^97^) has been reported previously.^98,99^ For less-accurate theoretical structures, with moiety RMSD values from 2.0 to 3.0 Å (*n* = 12), the R^2^ values of VC, GB, and PB all decreased sharply to 0.37/0.23, 0.35/0.32, and 0.32/0.28. For the least accurate poses (moiety RMSD values > 3.0 Å, *n* = 6), the correlation of the energy predictions for all three methods (Table S6) was completely abolished. Interestingly, interaction energies for single structures from energy minimization of the solvated crystallographic or GA-predicted complexes failed to result in acceptable correlations with experimental binding free energies (Table S6), indicating the importance of including a dynamic ensemble.

It is notable that positive theoretical interaction energies were obtained with both the MM-GBSA and PBSA methods for the calcium-dependent lectins: DC-SIGN, LecA, and LecB (Table S7). In these lectins, hydroxyl groups in the carbohydrate ligand coordinate to one or more calcium ions, whose presence is known to be essential for binding.^100^ The theoretical interaction energies were therefore in conflict with the experimental observation of the key role of calcium for promoting carbohydrate binding, and likely resulted from known weaknesses in the GBSA and PBSA formalisms when applied to calcium-mediated binding.^101–103^ An examination of the interaction energies associated with the calcium ions in these systems clearly illustrated the fundamental problem, namely that the unfavorable (positive) polar desolvation energies outweighed the favorable (negative) direct hydroxyl-ion electrostatic energies (ELE) obtained from the force field (Table S7). The magnitude of the repulsive interactions between the ligands and the calcium ions ranged from 5.8 ± 1.3 kcal/mol for PDB ID 6EYI (E-selectin complex) to 15.7 ± 2.9 for 8AIJ (LecB complex), with an average repulsion of almost 10 kcal/mol (Table S7). This issue has been attributed to limitations associated with the fixed partial charge models employed in such analyses.^104^ Despite the fact that MM-GBSA/PBSA failed to correctly predict the strength of calcium interactions, these lectins were retained in the Test data set because of their prevalence, reflecting their biological importance as therapeutic targets.^105–107^

Having established the performance of the glycomimetic design pipeline against a curated data set of 3D structures with corresponding binding energies, the protocol was then applied to a “real-world” set of glycomimetics with reported binding energies, but without 3D structures for their complexes (the “Application” data set, *n* = 73).

### Real-World Application

The preceding analyses employed a curated Test data set of high-resolution glycomimetic–protein complexes. However, in the initial stages of glycomimetic design, it is likely that only a structure of the endogenous carbohydrate-protein complex may be available. The Application data set was assembled to evaluate the performance of the proposed screening pipeline in such cases. Models of the glycomimetic complexes for the Application data set were created via the automated grafting of the glycomimetic moiety (from the moiety library) onto the endogenous carbohydrate bound to its receptor. As in the Test cases, the optimal orientation of the moiety was determined using the GA search algorithm, and the system subjected to MD simulation and energy evaluation (Figure 3, right column, Tables S8 and S9). The resulting interaction energies led to correlations with experimental values that were slightly degraded from those obtained for the most accurate Test structures (RMSD < 2 Å), but that followed a similar trend, with the MM-PBSA approach (R^2^ = 0.59) outperforming MM-GBSA (R^2^ = 0.55) and VC (R^2^ = 0.54). Interestingly, the inclusion of conformational entropies did not significantly improve the R^2^ values for the Application data set (Table S10). Regarding the three calcium-containing lectins in the Application data set, as in the Test data set, the VC energies were all negative, while MM-GBSA/PBSA energies were again positive. This issue was most pronounced for the LecB protein, which contains two calcium ions in the binding site.

### Induced Fit

The present protocol is intended to be applied to a complex of the endogenous ligand bound to its receptor, and any subsequent induced fit would therefore arise solely from the introduction of the new moiety. The effective treatment of induced fit in automated ligand docking remains a challenge.^108,109^ In the GA screening protocol, the proteins were treated as rigid, however, the simulation phase provided a mechanism to incorporate the effects of explicit solvent, as well as ligand and protein flexibility. The Test data set comprised glycomimetics crystallized in complex with the relevant receptors, and thus the protein structures in these systems included any conformational changes in the protein induced by the glycomimetic. Therefore, this data set enabled an assessment of the performance of the GA algorithm and energy analyses without concern for the need to consider induced fit. In contrast, the Application data set was selected to reflect the real-world case, in which a crystal structure of the glycomimetic–receptor complex may not exist. The crystal structures used in the Application data set did not contain the intact glycomimetic moiety, but only the endogenous carbohydrate. Thus, the conformations of the receptors in the Application data set were not necessarily perfectly complementary to the glycomimetic. For this reason, the modestly reduced performance when applied to the Application data set likely arose, at least in part, from induced fit that may not have been fully compensated for by post-GA MD simulation. As a specific example of the importance of induced fit, glycomimetic T32 (that binds to Galectin-3) was included in the Test data set (Table S1), and performed well (Table 3).

**Table 3:** Impact of Induced Fit on the Outcome (RMSD, Å) of GA Screening and Subsequent MD Simulation

initial ligand–receptor complex	GA moiety (Pre-MD)	ligand (Post-MD)	carbohydrate (Post-MD)	moiety (Post-MD)
Galectin-3: T32 (PDB ID 5E88)	0.07	0.96 ± 0.17	0.89 ± 0.27	1.01 ± 0.20
Galectin-3: endogenous carbohydrate (PDB ID 1KJL)	0.29	1.98 ± 0.17	0.84 ± 0.27	2.73 ± 0.20

However, the crystal structure of this glycomimetic complex (PDB ID 5E88, Figure 4) indicated that ligand binding induces a significant change in the conformation of residue R144, leading to its guanidino group sandwiching the thienyl ring of the glycomimetic against the protein surface.

**Figure 4:**
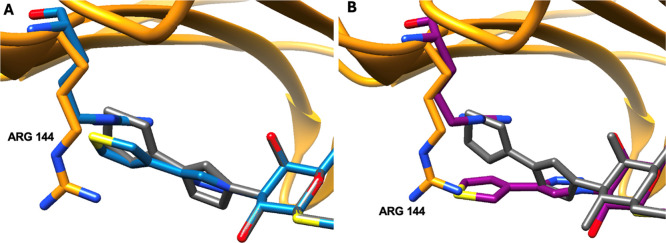
(A) Overlay of the moiety pose predicted by GA initiated with the endogenous carbohydrate in the binding site (PDB ID 1JKL, blue) and the experimental structure of the glycomimetic–Galectin-3 complex (PDB 5E88, orange for protein and gray for ligand), RMSD = 0.29 Å. (B) An overlay of the representative MD pose (purple) and PDB 5E88, RMSD = 1.98 Å.

When the GA/MD protocol was repeated for this glycomimetic, but employing the complex of the endogenous carbohydrate in the Galectin-3 binding site (PDB ID 1JKL) as the scaffold, the GA algorithm was unable to recapitulate the experimental orientation of the moiety (Figure 4). Although the moiety RMSD of the resulting GA structure for T32 was low (0.29 Å, Table 3), its orientation was such that the thienyl ring now sandwiched the side chain of R144 against the protein surface, in contrast to the relative orientations of the moiety and side chain in the experimental complex. Subsequent MD simulation (100 ns) was unable to correct these orientations (Table 3).

## Discussion

The approach described here was developed primarily for discerning likely conformations of bound glycomimetics, without focusing directly on improving the methods for binding energy evaluation. Nonetheless, it led to the identification of important systematic weaknesses in the VC, and MM-GBSA/PBSA approximations, some of which, such as the treatment of the *exo*-anomeric effect were corrected. Post-MD energy analysis with VC or MM-GBSA/PBSA offers a rapid method for high-throughput library screening. More theoretically rigorous approaches, such as thermodynamic integration (TI), have been compared to these simpler methods,^110^ and their relative utility in drug design reviewed.^111^ While TI has some benefits, especially as regards the explicit treatment of water-ligand interactions and entropy, it is extremely computationally demanding (at least relative to VC or MM-GBSA/PBSA), and achieving sampling convergence, particularly for larger moieties, would be very challenging. Lastly, the introduction of moieties that change the net charge of the system remain a challenge for perturbation approaches.^112^ By providing standard output formats, and curated structure and energy data sets, it is anticipated that the present work will facilitate future comparisons between theoretical methods.

The highest correlation with experimental binding energies, (R^2^ = 0.67) was achieved with the MM-PBSA approach, a value that is consistent with previous studies.^113–115^ Not surprisingly, the correlations were heavily dependent on the accuracy of the structural ensembles from the MD simulations. Although the average post-MD RMSD value of the moieties in the Test data set was modest at 2.5 ± 1.2 Å and independent of whether the simulations were initiated with the experimental crystal structures or with the structures from GA screening, there were systems that performed poorly. In addition to caveats associated with incomplete treatment of induced fit, two other factors deserve consideration. First, all of the present MD simulations employed the widely used TIP3P^116^ water model. However, its simple electrostatic description detracts from the ability of TIP3P to reproduce water-mediated carbohydrate-protein interactions.^117^ Crystallographic waters that interact simultaneously with the ligand and the receptor are ubiquitous in carbohydrate-protein complexes, and the choice of water model for the MD simulation can be critical for reproduction of the binding mode.^117^ Poor treatment of such interactions may be the basis for the relatively large moiety RMSD values seen, for example, in MD simulations of the T5- and T51-receptor complexes (Figure 5).

**Figure 5:**
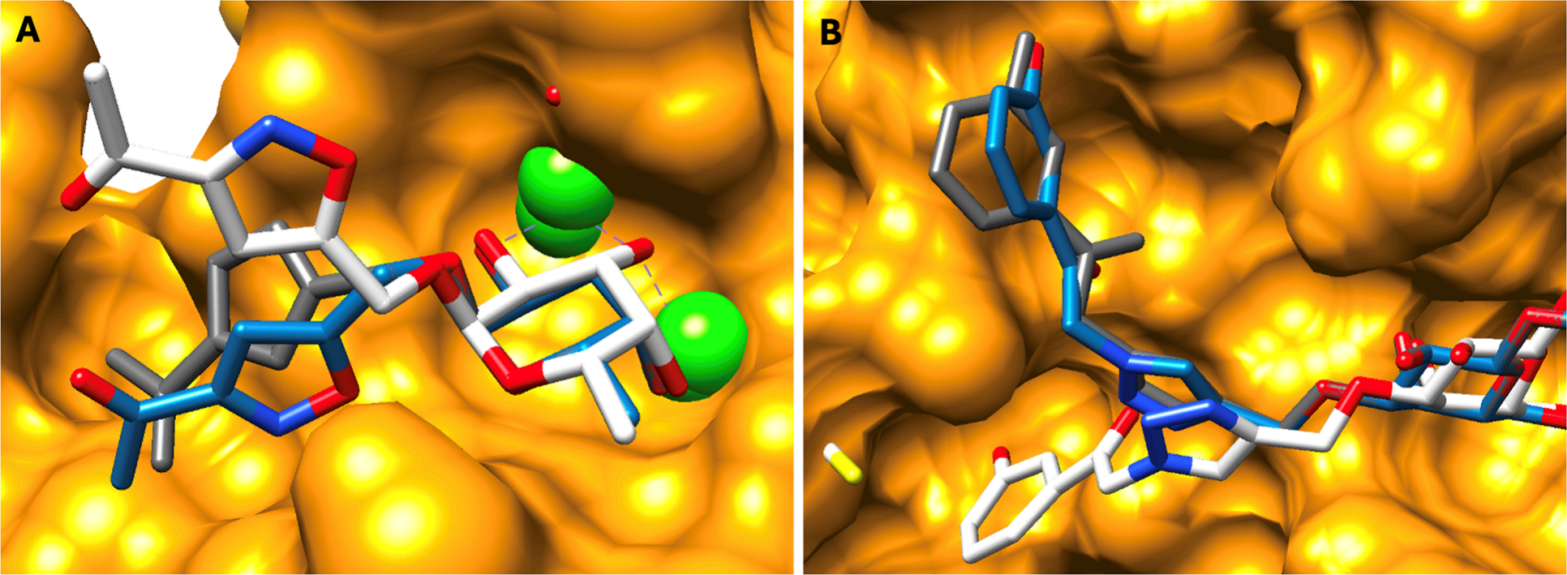
Two glycomimetics ((A) T5, PDB ID 2VUC (LecB) and (B) T51, PDB ID 4Q1R (Galectin-1)) with the largest post-MD moiety RMSD values from the Test data set. Blue, structure from GA screening; white, the representative MD frame; and orange and gray, protein and ligand experimental structure, respectively. Calcium ions are shown as green spheres.

Second, force field limitations may impact the accuracy of the conformation and interaction energies of the ligands. Although the core carbohydrate residues consistently remained stable during MD simulations, variations in the moiety poses were sometimes significant. Many of the crystal structures indicated the presence of CH−π interactions between the glycomimetic moiety and the protein surface residues. Such interactions are not explicitly parametrized into the AMBER force field, and have been shown to be only partially accounted for by van der Waals forces.^118^ Poor treatment of these interactions could destabilize the moiety in the binding site, and could attenuate the ability of MD simulations to reproduce induced fit in the protein surface. Differences between the experimental and MD-generated poses of T5 and T51 may also have resulted from weaknesses in the general AMBER force field (GAFF) employed for the moieties or from limitations in the treatment of electrostatic interactions, particularly with calcium-containing lectins.

The present work reports the creation, validation, and dissemination of a novel computational pipeline designed with the goal of enabling automation in rational mimetic discovery,^6,119,120^ in the hope that it may expedite the prioritization of experimental efforts to accelerate the development of this broadly relevant class of therapeutics.

## Supplementary Material





## Data Availability

Source code and moiety libraries (1543 PDBQT files) are distributed as a GIT repository (https://github.com/GLYCAM-Web/glycomimetics) free of charge. Instructions for compilation and the execution of an example run are provided in the repository. The C++ programs developed in this work take advantage of the GLYCAM Molecular Modeling Library (GMML), a C++ functional library developed in the Woods group and distributed as a GIT repository (https://github.com/GLYCAM-Web/gmml2) free of charge. Instructions for installation of GMML are provided in the repository.

## Notes

Figures S1–S5 and Tables S1–S10 (PDF)
All PDBQT files for the protein-carbohydrate complexes, all PDB files corresponding to the output of GA search, and all plain text log files corresponding to the output of PDB2GLYCAM (ZIP)
