# Supplementary material for: Computational Pipeline for Accelerating the Design of Glycomimetics
Source: J Chem Inf Model. 2025 Nov 27;65(24):13374–85. doi: 10.1021/acs.jcim.5c02282 (PMC12728930; doi:10.1021/acs.jcim.5c02282)
Supplement: Supplementary file 1 [file ci5c02282_si_001.pdf]

Supporting Information for

A Computational Pipeline for Accelerating the Design of Glycomimetics

*Yao Xiao, Alexander H. Lee, Sawsan Mahmoud, Bilqees Sameem, Daniel Wentworth, Xiaocong Wang, Grayson D. Miller, Oliver C. Grant, B. Lachele Foley, Robert J. Woods**

*Robert J. Woods**

Email: rwoods@ccrc.uga.edu

This PDF file includes:

Supporting text

Figures S1 to S5

Tables S1 to S10

Materials and Methods

Curated glycomimetic-receptor datasets.

These structural and energetic criteria led to the acceptance of 58 protein-glycomimetic systems (glycomimetic structures T1-T58, Table S1) that included the following ten carbohydrate receptor proteins: CT (1-3), DC-SIGN (4-6), FimH (7-10), FmlH (11), Galectin-1 (12-15), Galectin-3 (16-22), LecA (23-26), LecB (27-31), Siglec-7 (32), and E-selectin (33, 34).

The resulting Application dataset comprised 73 glycomimetic ligands that were not included in the Test dataset, but that targeted some of the same receptor proteins: DC-SIGN (4), galectin-1 and 3 (21), LecA (35), and LecB (27) (Table S7, A1-A73).

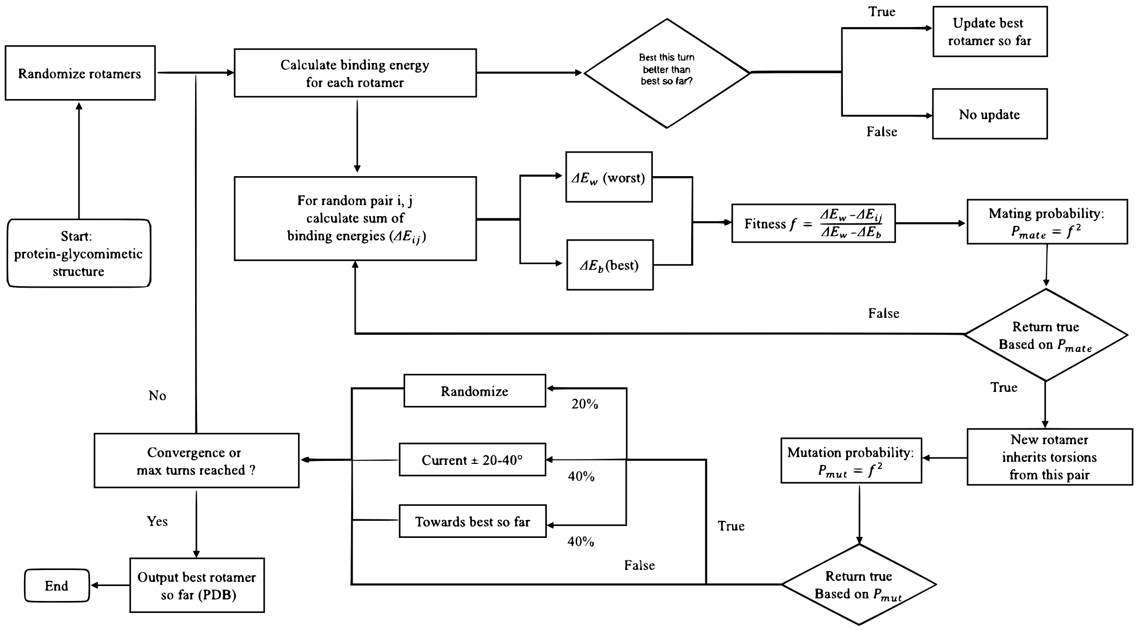

Figure S1. Genetic algorithm for conformational sampling of moiety orientations.

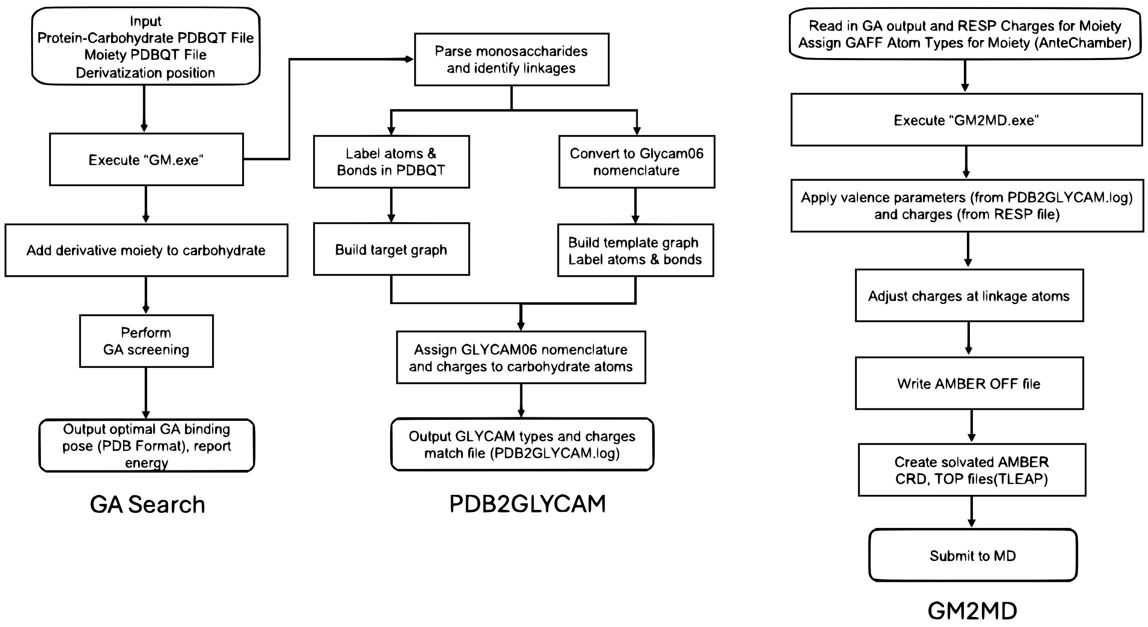

Figure S2. Overall GM workflow.

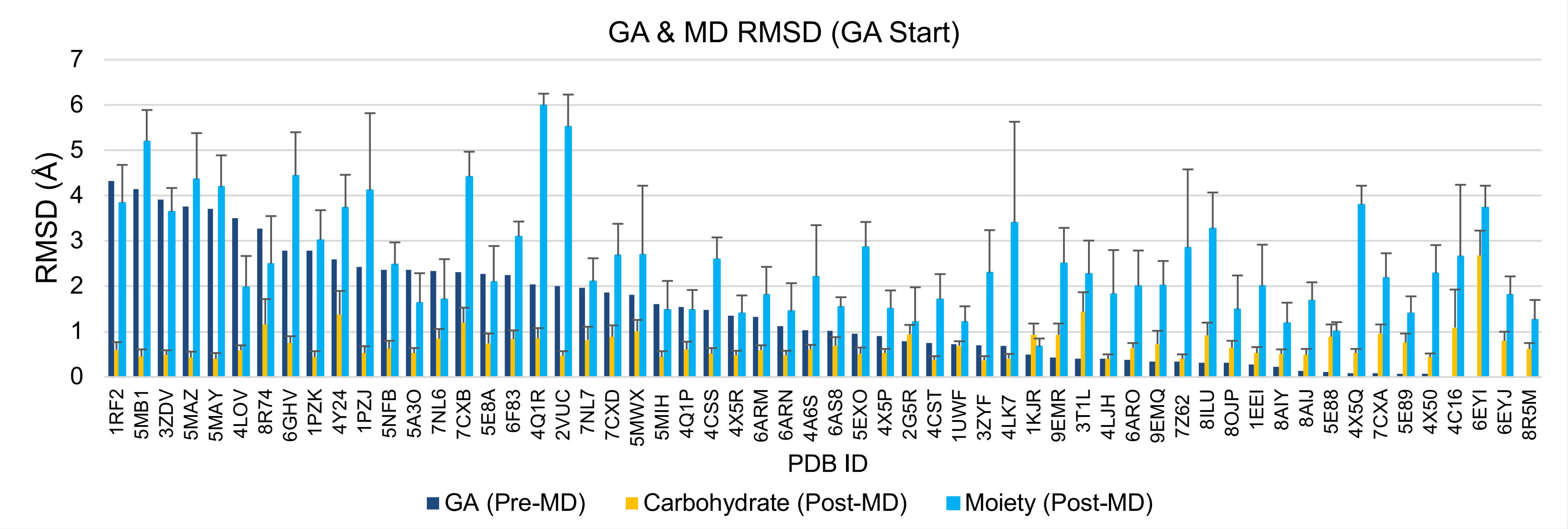

Figure S3. RMSD values from GA scanning and MD simulations (initiated from GA-predicted moiety poses) of mimetics in the Test dataset relative to the experimental structures. Horizontal axis: PDB ID. Vertical axis: RMSD (Å). Error bars represent standard deviation values.

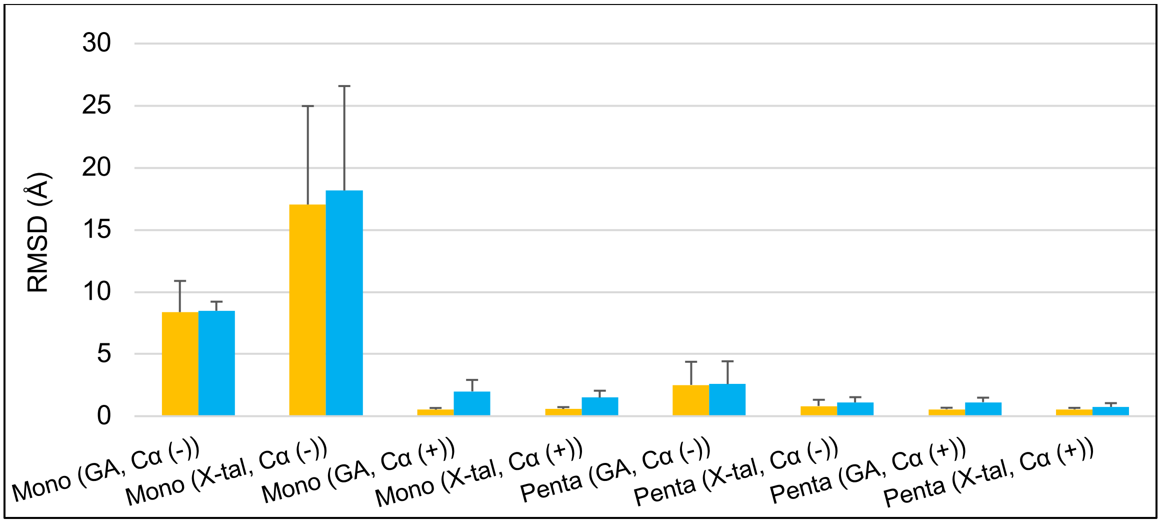

Figure S4. Post-MD RMSD values for CT glycomimetic T41 (PDB ID 1EEI) as a function of simulation condition for the carbohydrate only (yellow) and the moiety only (cyan). The MD simulations were performed either with or without Cα restraints, Cα (+) and Cα (-), respectively, applied to the monomeric (Mono) or pentameric (Penta) protein structures. The simulations were initiated with the ligand in the experimental pose (X-tal) or with the pose from the GA screening (GA). Error bars represent the standard deviations over the MD trajectories.

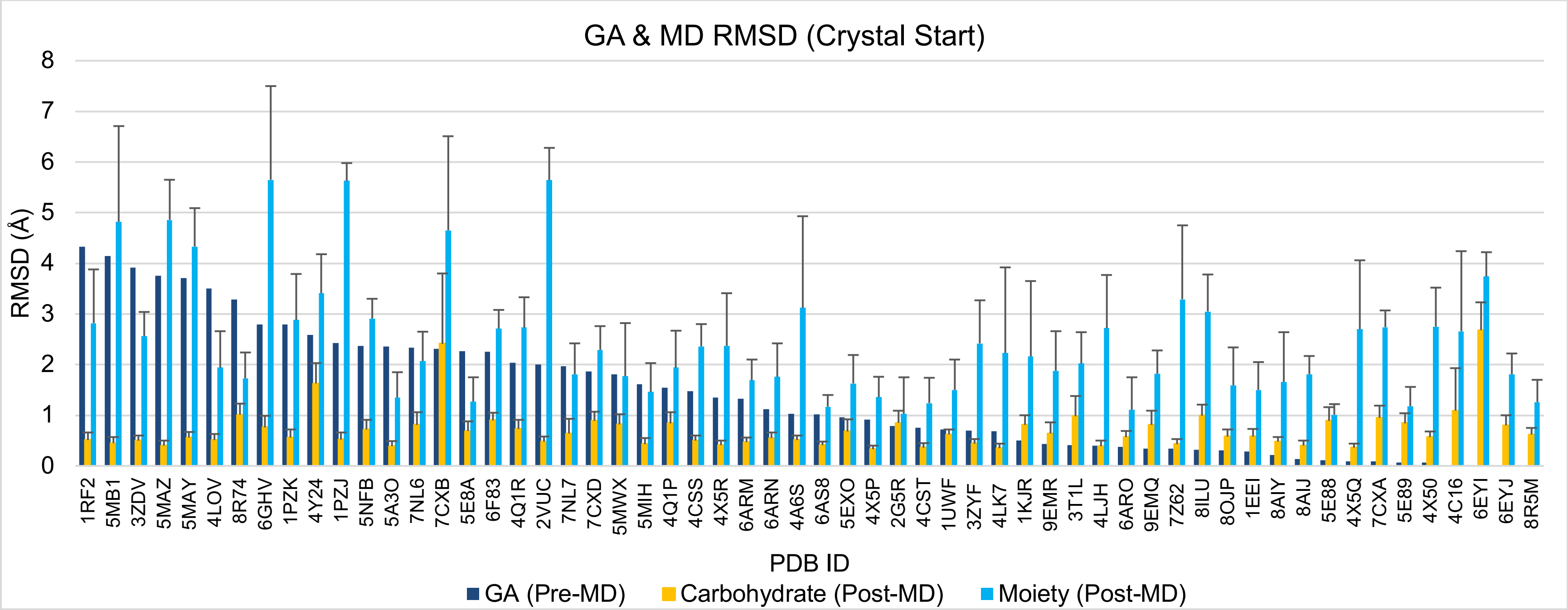

Figure S5. RMSD values from GA scanning and MD simulations (initiated from crystallographic coordinates) of mimetics in the Test dataset relative to the crystal structure. Horizontal axis: PDB ID. Vertical axis: RMSD (Å). Error bars represent standard deviation values.

Table S1. PDB structures in the glycomimetic Test dataset.

PDB ID	Resolution (Å)	Average Protein B-Factor (Å^2)	Average Ligand B-Factor (Å^2)	Chain ID	MD Waters	Retained Crystallographic Water Number(s)	Experimental Affinity Method	Glycomimetic Identifier
5A3O	1.60	8.3	8.3	B	5495	484, 485, 486, 495	FP	T1
1UWF	1.69	13.0	11.0	A	10049	2255, 2269, 2271, 2302	ITC	T2
4LOV	1.50	12.0	11.2	A	10331	None	ITC	T3
4X5R	1.65	14.9	11.3	A	10510	619	ITC	T4
2VUC	1.30	6.3	11.6	B	4993	2161	N/A	T5
5MAZ	1.45	10.4	12.3	C	5393	None	ITC	T6
3ZDV	1.41	12.8	12.7	C	5170	484, 485, 486, 495	FP	T7
5MB1	1.65	6.7	14.0	A	5513	None	ITC	T8
4X50	2.00	17.5	14.2	A	10435	429, 449, 532, 560, 570, 572, 578, 579	ITC	T9
6ARO	1.20	14.5	14.9	A	8627	330, 390	BLI	T10
4CST	1.10	12.4	15.3	A	9487	2001	FP	T11
5MAY	1.65	9.9	15.8	A	5051	None	ITC	T12
4X5Q	1.12	14.2	15.9	A	10158	658	ITC	T13
4X5P	1.00	13.6	16.4	A	10442	532	ITC	T14
5MIH	1.80	15.4	17.1	A	6181	344	FP	T15
4CSS	1.07	12.6	17.4	A	10413	None	FP	T16
4LJH	1.45	19.7	17.7	A	5840	1004	ITC	T17
7CXB	1.46	23.9	18.4	A	6399	433, 468, 474	ITC	T18
8AIJ	1.50	13.9	18.8	A	5870	None	ITC	T19
8ILU	1.80	20.8	18.9	A	6859	401, 412, 447, 449	FP	T20
6ARN	1.25	20.5	19.9	B	8628	330, 390	BLI	T21
8AIY	1.55	21.0	20.6	A	5231	None	ITC	T22
1RF2	1.35	11.7	21.6	All	9942	None	ELISA	T22

1RF2	1.35	11.4	21.6	D	4325	None	ELISA	T23
7Z62	1.53	16.6	21.6	A	6078	345	ITC	T24
1KJR	1.55	15.4	22.3	A	5548	691	FP	T25
5E89	1.50	14.4	23.4	A	5443	456, 475	FP	T26
5NFB	1.59	15.9	24.3	A	4969	404, 456, 501	ITC	T27
8OJP	1.71	36.9	24.8	A	4470	319, 324, 343, 402	FP	T28
4LK7	1.76	23.9	25.9	A	5862	1007	ITC	T29
5EXO	1.50	15.5	26.6	A	5002	420, 442, 488	N/A	T30
6ARM	1.50	21.4	27.7	A	8625	330, 390	BLI	T31
5E88	1.60	16.1	28.6	A	5347	None	FP	T32
7NL6	2.20	32.8	29.1	A	6547	None	ITC	T33
9EMR	1.90	26.3	29.2	A	7770	None	ITC	T34
8R74	1.54	29.7	29.6	A	4871	615	FP	T35
5E8A	1.50	15.5	29.7	A	5001	456, 475	FP	T36
3T1L	1.60	17.7	29.8	A	5305	84, 90, 91	N/A	T37
7CXD	1.71	30.0	30.1	A	4767	437	ITC	T38
9EMQ	1.80	22.8	30.2	A	8737	None	ITC	T39
1PZK	1.35	8.0	30.6	All	10144	1576, 1577, 1586 (chain D)	ELISA	T40
1PZK	1.35	7.8	30.6	D	4413	1576, 1577, 1586	ELISA	T40
1EEI	2.00	27.8	31.7	D	4258	520, 527, 533, 537	ELISA	T41
1EEI	2.00	25.1	31.7	All	9872	520, 527, 533, 537 (chain D)	ELISA	T41
1PZJ	1.46	11.8	32.6	All	10106	1513, 1515, 1516, 1520 (chain D)	ELISA	T42
1PZJ	1.46	10.8	32.6	D	4674	1513, 1515, 1516, 1520	ELISA	T42
4Q1P	1.46	32.1	35.1	A	4409	319, 349	ITC	T43
7NL7	2.10	36.7	37.2	A	6664	None	ITC	T44
3ZYF	1.94	27.7	37.5	A	5698	2054, 2081	ITC	T45
6GHV	2.10	34.8	39.6	A	8594	None	ITC	T46

6AS8	2.10	32.5	39.8	A	9015	305, 309	BLI	T47
4A6S	2.15	40.4	42.3	A	5676	2054, 2081	ITC	T48
2G5R	1.60	17.8	44.0	A	6539	1088	ELISA	T49
5MWX	1.29	35.0	44.2	A	4493	349	ITC	T50
4Q1R	1.47	36.7	44.6	A	4427	None	ITC	T51
4Y24	2.32	32.5	45.3	A	4536	None	ITC	T52
4C16	1.93	40.2	53.1	A	4397	None	ITC	T53
6F83	2.20	37.4	55.3	A	4526	None	FP	T54
7CXA	1.97	65.2	67.6	A	5240	None	ITC	T55
6EYJ	2.20	67.0	81.4	A	4205	None	MST	T56
6EYI	2.04	71.4	84.7	A	4464	None	MST	T57
8R5M	2.49	74.0	93.2	A	4150	None	ITC	T58

Table S2. Ligand binding energies (kcal/mol) for the Test set computed with VC, MM-GBSA, and MM-PBSA.

Receptor	PDB	Experimental	GA	MD			N_{rot} [a]	$-T\Delta S_{lig}$	Total Binding Energy		
			VC	VC	MM/GBSA	MM/PBSA			ΔG_{VC}	ΔG_{GBSA}	ΔG_{PBSA}
DC-SIGN	6GHV	-5.9	-9.9	-8.7 ± 1.0	-18.7 ± 4.2	-2.7 ± 4.5	9	5.4	-3.3	-13.3	2.7
	9EMQ	-6.0	-8.4	-7.1 ± 0.6	-21.9 ± 4.8	-10.1 ± 7.0	1	0.6	-6.5	-21.3	-9.5
	9EMR	-5.3	-8.3	-6.2 ± 0.6	-10.5 ± 4.6	-1.6 ± 6.4	2	1.2	-5.0	-9.3	-0.4
	7NL6	-5.2	-6.2	-5.0± 0.4	-6.6 ± 3.4	1.0 ± 4.0	0	0.0	-5.0	-6.6	1.0
	7NL7	-6.1	-7.1	-6.1 ± 0.6	-17.3 ± 3.7	-7.9 ± 4.8	2	1.2	-4.9	-16.1	-6.7
FimH	4CSS	-11.5	-10.0	-10.5 ± 0.7	-57.7 ± 3.1	-37.0 ± 4.3	2	1.2	-9.3	-56.5	-35.8
	4CST	-12.1	-11.6	-10.3 ± 0.7	-57.1 ± 2.8	-35.1 ± 3.3	1	0.6	-9.7	-56.5	-34.5
	4LOV	-10.6	-9.3	-9.4 ±0.7	-58.5 ± 3.3	-40.2 ± 3.7	5	3.0	-6.4	-55.5	-37.2
	4X50	-10.6	-11.5	-9.6 ± 0.7	-54.7 ± 2.9	-32.8 ± 3.1	0	0.0	-9.6	-54.7	-32.8
	4X5Q	-12.3	-11.5	-10.6 ± 0.8	-57.4 ± 2.9	-32.6 ± 3.2	0	0.0	-10.6	-57.4	-32.6
	1UWF	-9.4	-9.4	-7.9 ± 0.4	-51.5 ± 2.8	-31.6 ± 3.1	2	1.2	-6.7	-50.3	-30.4
	4X5P	-11.2	-10.5	-10.2 ± 0.7	-57.4 ± 2.8	-36.9 ± 3.3	0	0.0	-10.2	-57.4	-36.9
	4X5R	-10.7	-10.6	-9.8 ± 0.6	-59.3 ± 3.2	-34.9 ± 3.8	3	1.8	-8.0	-57.5	-33.1
Gal-1	8OJP	-8.6	-8.8	-8.7 ± 0.5	-36.0 ± 2.7	-6.3 ± 3.7	1	0.6	-8.1	-35.4	-5.7
	4Q1P	-5.6	-11.1	-10.1 ± 0.5	-40.5 ± 3.0	-11.2 ± 3.8	4	2.4	-7.7	-38.1	-8.8
	4Q1R	-5.5	-10.7	-10.7 ± 0.6	-42.3 ± 3.1	-10.6 ± 4.0	4	2.4	-8.3	-39.9	-8.2
	5MWX	-5.4	-10.1	-8.5 ± 1.3	-39.4 ± 4.6	-8.3 ± 4.7	5	3.0	-5.5	-36.4	-5.3
	4Y24	-9.1	-12.2	-11.9 ± 1.5	-45.2 ± 5.3	-16.8 ± 4.3	0	0.0	-11.9	-45.2	-16.8
	6F83	-11.2	-11.3	-11.6 ± 0.7	-48.4 ± 3.6	-20.0 ± 4.6	0	0.0	-11.6	-48.4	-20.0
	8R74	-8.8	-8.4	-7.0 ± 0.9	-26.8 ± 4.7	0.5 ± 4.5	1	0.6	-6.4	-26.2	1.1
Gal-3	5E89	-10.7	-12.0	-12.4 ± 1.0	-46.7 ± 3.2	-22.7 ± 3.4	0	0.0	-12.4	-46.7	-22.7
	5E8A	-10.2	-14.8	-12.4 ± 1.0	-44.8 ± 3.3	-21.2 ± 3.5	0	0.0	-12.4	-44.8	-21.2
	5E88	-9.8	-12.2	-10.9 ± 0.7	-53.1 ± 3.1	-30.3 ± 3.6	0	0.0	-10.9	-53.1	-30.3
	7CXA	-11.1	-13.2	-12.6 ± 1.1	-47.2 ± 3.2	-24.4 ± 3.5	0	0.0	-12.6	-47.2	-24.4

	7CXB	-9.4	-12.4	-10.4 ± 1.1	-42.5 ± 3.9	-20.5 ± 3.9	0	0.0	-10.4	-42.5	-20.5
	7CXD	-8.2	-11.5	-10.6 ± 0.8	-39.2 ± 3.3	-15.3 ± 3.7	0	0.0	-10.6	-39.2	-15.3
	1KJR	-8.3	-12.4	-10.2 ± 0.7	-38.3 ± 3.1	-15.4 ± 3.7	1	0.6	-9.6	-37.7	-14.8
	5NFB	-8.3	-11.5	-10.7 ± 0.9	-39.7 ± 3.7	-14.7 ± 4.1	3	1.8	-8.9	-37.9	-12.9
	8ILU	-9.8	-11.7	-7.3 ± 1.0	-21.7 ± 4.5	1.9 ± 4.7	2	1.2	-6.1	-20.5	3.1
LecA	5MIH	-5.7	-8.0	-6.0 ± 0.4	-8.4 ± 3.4	0.1 ± 3.4	0	0.0	-6.0	-8.4	0.1
	7Z62	-7.5	-9.3	-6.9 ± 0.5	-10.6 ± 3.9	0.6 ± 3.8	4	2.4	-4.5	-8.2	3.0
	4LJH	-6.4	-8.3	-6.9 ± 0.4	-10.1 ± 3.7	-1.4 ± 3.6	0	0.0	-6.9	-10.1	-1.4
	4LK7	-6.9	-8.8	-6.8 ± 0.5	-9.6 ± 3.7	0.4 ± 3.8	0	0.0	-6.8	-9.6	0.4
	3ZYF	-6.6	-6.3	-6.4 ± 0.4	-6.8 ± 3.6	1.2 ± 3.6	1	0.6	-5.8	-6.2	1.8
	4A6S	-7.1	-6.3	-6.5 ± 0.4	-9.1 ± 3.4	-0.7 ± 3.3	1	0.6	-5.9	-8.5	-0.1
LecB	5MB1	-8.9	-10.0	-6.6 ± 0.7	1.0 ± 4.7	10.9 ± 4.6	3	1.8	-4.8	2.8	12.7
	5MAY	-8.9	-8.8	-5.7 ± 0.4	3.4 ± 4.3	5.8 ± 4.3	3	1.8	-3.9	5.2	7.6
	8AIJ	-7.7	-6.9	-5.8 ± 0.4	5.6 ± 4.2	8.0 ± 3.9	0	0.0	-5.8	5.6	8.0
	8AIY	-9.6	-7.4	-8.1 ± 0.5	-3.4 ± 4.2	3.8 ± 3.8	0	0.0	-8.1	-3.4	3.8
	5MAZ	-8.8	-9.1	-6.4 ± 0.6	0.1 ± 4.6	6.5 ± 4.4	3	1.8	-4.6	1.9	8.3
	3ZDV	-7.5	-10.6	-6.5 ± 0.6	3.6 ± 4.4	9.9 ± 3.5	2	1.2	-5.3	4.8	11.1
	5A3O	-6.0	-9.1	-6.6 ± 0.6	1.8 ± 4.0	5.4 ± 3.5	0	0.0	-6.6	1.8	5.4
E-selectin	6EYJ	-6.3	-11.5	-11.5 ± 0.7	-38.6 ± 5.4	-10.4 ± 6.7	8	4.8	-6.7	-33.8	-5.6
	6EYI	-5.7	-11.4	-7.9 ± 0.7	-26.9 ± 4.8	-6.0 ± 6.6	8	4.8	-3.1	-22.1	-1.2
	4C16	-6.5	-11.5	-10.4 ± 0.9	-41.8 ± 6.2	-17.3 ± 6.4	8	4.8	-5.6	-37.0	-12.5
	8R5M	-7.8	-12.5	-11.4 ± 0.6	-44.8 ± 4.7	-20.4 ± 6.3	11	6.6	-4.8	-38.2	-13.8

[a]Number of bonds treated as rotatable in the moiety.

Table S3. RMSD[a] values (Å) from GA scanning and MD simulations (initiated from the GA-predicted structures).

Receptor	PDB ID	Moiety Only GA (Pre-MD)	Whole Ligand (Post-MD)		Carbohydrate Only (Post-MD)		Moiety Only (Post-MD)	
			Average	Standard Deviation	Average	Standard Deviation	Average	Standard Deviation
Cholera	1RF2	4.33	2.76	0.58	0.60	0.17	3.84	0.84
LecB	5MB1	4.14	3.98	0.52	0.45	0.16	5.20	0.69
LecB	3ZDV	3.91	2.70	0.39	0.49	0.10	3.64	0.53
LecB	5MAZ	3.76	3.23	0.75	0.43	0.13	4.36	1.02
LecB	5MAY	3.71	2.98	0.49	0.40	0.13	4.19	0.70
FimH	4LOV	3.50	1.30	0.40	0.59	0.11	1.98	0.69
Galectin-1	8R74	3.28	2.15	0.89	1.16	0.56	2.49	1.06
DC-SIGN	6GHV	2.79	3.96	0.85	0.75	0.15	4.44	0.96
Cholera	1PZK	2.79	2.01	0.42	0.44	0.13	3.02	0.66
Galectin-1	4Y24	2.59	2.75	0.58	1.38	0.52	3.73	0.73
Cholera	1PZJ	2.43	3.40	1.40	0.53	0.15	4.12	1.70
Galectin-3	5NFB	2.37	1.87	0.35	0.63	0.17	2.48	0.49
LecB	5A3O	2.36	1.23	0.44	0.53	0.11	1.64	0.65
DC-SIGN	7NL6	2.34	1.37	0.56	0.85	0.21	1.71	0.89
Galectin-3	7CXB	2.31	3.37	0.42	1.20	0.33	4.42	0.55
Galectin-3	5E8A	2.27	1.65	0.55	0.74	0.22	2.10	0.79
Galectin-1	6F83	2.25	2.26	0.22	0.84	0.19	3.10	0.33
Galectin-1	4Q1R	2.04	3.95	0.16	0.85	0.23	5.99	0.26
LecB	2VUC	2.00	3.72	0.48	0.47	0.10	5.52	0.71
DC-SIGN	7NL7	1.97	1.70	0.39	0.82	0.29	2.11	0.51
Galectin-3	7CXD	1.86	2.08	0.50	0.89	0.25	2.68	0.70
Galectin-1	5MWX	1.81	1.96	0.97	1.01	0.25	2.70	1.52
LecA	5MIH	1.61	1.08	0.41	0.44	0.13	1.48	0.64
Galectin-1	4Q1P	1.55	1.04	0.26	0.61	0.17	1.48	0.44
FimH	4CSS	1.48	2.00	0.36	0.52	0.12	2.60	0.48

FimH	4X5R	1.35	1.16	0.31	0.48	0.10	1.41	0.39
FmlH	6ARM	1.33	1.28	0.39	0.59	0.11	1.81	0.62
FmlH	6ARN	1.12	1.00	0.37	0.48	0.10	1.45	0.62
LecA	4A6S	1.03	1.63	0.79	0.61	0.10	2.21	1.14
FmlH	6AS8	1.02	1.16	0.15	0.69	0.19	1.54	0.22
Galectin-3	5EXO	0.96	2.13	0.40	0.51	0.14	2.87	0.55
FimH	4X5P	0.91	1.23	0.30	0.53	0.09	1.51	0.40
Siglec-7	2G5R	0.79	1.04	0.31	0.94	0.21	1.21	0.77
FimH	4CST	0.75	1.30	0.41	0.38	0.08	1.71	0.56
FimH	1UWF	0.72	0.86	0.16	0.69	0.10	1.21	0.35
LecA	3ZYF	0.70	1.61	0.64	0.37	0.09	2.30	0.94
LecA	4LK7	0.69	2.56	1.64	0.41	0.10	3.40	2.23
Galectin-3	1KJR	0.50	0.85	0.19	0.93	0.25	0.67	0.18
DC-SIGN	9EMR	0.43	2.03	0.58	0.93	0.25	2.51	0.78
Galectin-3	3T1L	0.41	1.90	0.55	1.44	0.43	2.27	0.74
LecA	4LJH	0.40	1.28	0.64	0.40	0.10	1.83	0.97
FmlH	6ARO	0.38	1.44	0.50	0.64	0.11	2.00	0.79
DC-SIGN	9EMQ	0.34	1.66	0.43	0.73	0.29	2.02	0.54
LecA	7Z62	0.34	2.24	1.35	0.40	0.10	2.85	1.73
Galectin-3	8ILU	0.32	2.86	0.69	0.92	0.28	3.27	0.80
Galectin-1	8OJP	0.31	1.27	0.57	0.65	0.15	1.49	0.75
Cholera	1EEI	0.28	1.38	0.59	0.53	0.13	2.00	0.92
LecB	8AIY	0.22	0.99	0.32	0.51	0.10	1.19	0.45
LecB	8AIJ	0.13	1.22	0.27	0.49	0.13	1.69	0.40
Galectin-3	5E88	0.11	0.96	0.17	0.89	0.27	1.01	0.20
FimH	4X5Q	0.09	2.96	0.32	0.53	0.09	3.80	0.42
Galectin-3	7CXA	0.09	1.76	0.36	0.96	0.20	2.19	0.54
Galectin-3	5E89	0.07	1.17	0.26	0.76	0.20	1.41	0.37
FimH	4X50	0.07	1.65	0.43	0.44	0.08	2.29	0.62
E-selectin	4C16	0.00	2.03	1.23	1.09	0.84	2.66	1.58
E-selectin	6EYI	0.00	3.26	0.47	2.68	0.55	3.74	0.48

E-selectin	6EYJ	0.00	1.43	0.31	0.80	0.20	1.81	0.41
E-selectin	8R5M	0.00	1.05	0.32	0.62	0.13	1.26	0.44
Average ± one standard deviation		1.51 ± 1.22	1.95 ± 0.88		0.72 ± 0.36		2.55 ± 1.20	

[a]Relative to the ligand in the pertinent glycomimetic crystal structure.

Table S4. RMSD[a] values (Å) of GA scanning and MD simulations (initiated from the crystal structures).

Receptor	PDB ID	Moiety Only GA (Pre-MD)	Whole Ligand (Post-MD)		Carbohydrate Only (Post-MD)		Moiety Only (Post-MD)	
			Average	Standard Deviation	Average	Standard Deviation	Average	Standard Deviation
Cholera	1RF2	4.33	2.03	0.74	0.51	0.15	2.82	1.06
LecB	5MB1	4.14	3.71	1.42	0.45	0.12	4.82	1.89
LecB	3ZDV	3.91	1.92	0.34	0.50	0.10	2.56	0.48
LecB	5MAZ	3.76	3.60	0.58	0.40	0.10	4.86	0.79
LecB	5MAY	3.71	3.09	0.53	0.56	0.11	4.33	0.76
FimH	4LOV	3.50	1.25	0.42	0.51	0.12	1.94	0.72
Galectin-1	8R74	3.28	1.54	0.41	1.01	0.22	1.73	0.51
DC-SIGN	6GHV	2.79	5.03	1.64	0.76	0.23	5.65	1.85
Cholera	1PZK	2.79	1.94	0.58	0.56	0.16	2.88	0.91
Galectin-1	4Y24	2.59	2.63	0.55	1.63	0.40	3.41	0.77
Cholera	1PZJ	2.43	4.64	0.29	0.52	0.14	5.63	0.35
Galectin-3	5NFB	2.37	2.19	0.28	0.72	0.19	2.91	0.39
LecB	5A3O	2.36	1.00	0.34	0.39	0.10	1.35	0.50
DC-SIGN	7NL6	2.34	1.56	0.39	0.81	0.25	2.07	0.58
Galectin-3	7CXB	2.31	3.82	0.62	2.41	1.39	4.65	1.86
Galectin-3	5E8A	2.27	1.06	0.34	0.69	0.19	1.27	0.48
Galectin-1	6F83	2.25	2.00	0.25	0.90	0.15	2.71	0.37
Galectin-1	4Q1R	2.04	1.87	0.38	0.73	0.18	2.73	0.60
LecB	2VUC	2.00	3.80	0.43	0.48	0.10	5.64	0.64
DC-SIGN	7NL7	1.97	1.45	0.46	0.64	0.29	1.81	0.61
Galectin-3	7CXD	1.86	1.80	0.33	0.89	0.18	2.29	0.47
Galectin-1	5MWX	1.81	1.34	0.67	0.82	0.20	1.77	1.05
LecA	5MIH	1.61	1.06	0.37	0.43	0.12	1.46	0.57
Galectin-1	4Q1P	1.55	1.38	0.43	0.84	0.22	1.94	0.73
FimH	4CSS	1.48	1.82	0.32	0.50	0.10	2.36	0.44

FimH	4X5R	1.35	1.91	0.82	0.41	0.09	2.37	1.04
FmlH	6ARM	1.33	1.17	0.26	0.47	0.09	1.69	0.41
FmlH	6ARN	1.12	1.20	0.40	0.55	0.11	1.76	0.66
LecA	4A6S	1.03	2.25	1.26	0.51	0.09	3.12	1.81
FmlH	6AS8	1.02	0.88	0.16	0.41	0.07	1.17	0.23
Galectin-3	5EXO	0.96	1.29	0.42	0.68	0.24	1.63	0.56
FimH	4X5P	0.91	1.08	0.31	0.33	0.07	1.36	0.40
Siglec-7	2G5R	0.79	0.92	0.32	0.85	0.24	1.03	0.72
FimH	4CST	0.75	0.96	0.36	0.36	0.09	1.24	0.50
FimH	1UWF	0.72	0.94	0.26	0.62	0.10	1.50	0.60
LecA	3ZYF	0.70	1.71	0.57	0.44	0.09	2.42	0.85
LecA	4LK7	0.69	1.69	1.24	0.35	0.09	2.23	1.69
Galectin-3	1KJR	0.50	1.53	0.83	0.81	0.19	2.16	1.49
DC-SIGN	9EMR	0.43	1.52	0.59	0.64	0.22	1.88	0.78
Galectin-3	3T1L	0.41	1.58	0.47	0.98	0.40	2.02	0.62
LecA	4LJH	0.40	1.86	0.70	0.39	0.11	2.72	1.05
FmlH	6ARO	0.38	0.88	0.40	0.57	0.12	1.11	0.64
DC-SIGN	9EMQ	0.34	1.53	0.34	0.81	0.28	1.82	0.46
LecA	7Z62	0.34	2.58	1.13	0.43	0.10	3.29	1.46
Galectin-3	8ILU	0.32	2.68	0.62	0.99	0.22	3.05	0.73
Galectin-1	8OJP	0.31	1.34	0.57	0.58	0.14	1.59	0.75
Cholera	1EEI	0.28	1.09	0.34	0.58	0.15	1.50	0.55
LecB	8AIY	0.22	1.33	0.75	0.48	0.09	1.66	0.98
LecB	8AIJ	0.13	1.28	0.24	0.40	0.10	1.81	0.36
Galectin-3	5E88	0.11	0.96	0.22	0.89	0.27	1.01	0.21
FimH	4X5Q	0.09	2.11	1.04	0.36	0.08	2.70	1.36
Galectin-3	7CXA	0.09	2.13	0.23	0.95	0.24	2.74	0.33
Galectin-3	5E89	0.07	1.05	0.26	0.84	0.20	1.18	0.38
FimH	4X50	0.07	1.99	0.54	0.57	0.11	2.75	0.77
E-selectin	4C16	0.00	2.03	1.23	1.09	0.84	2.66	1.58
E-selectin	6EYI	0.00	3.26	0.47	2.68	0.55	3.74	0.48

E-selectin	6EYJ	0.00	1.43	0.31	0.80	0.20	1.81	0.41
E-selectin	8R5M	0.00	1.05	0.32	0.62	0.13	1.26	0.44
Average ± one standard deviation		1.51 ± 1.22	1.87 ± 0.95		0.71 ± 0.42		2.44 ± 1.19	

[a]Relative to the pertinent glycomimetic crystal structure.

Table S5. Correlation coefficients (R^2) between computed and experimental binding energies as a function of ligand conformational entropies ($-T\Delta S_{lig}$) per rotatable bond, for structures in the Test dataset with post-MD RMSD < 2.0 Å (n=28).

**$-T\Delta S_{lig}$ Per Bond**	**MD VC**	**MD MM-GBSA**	**MD MM-PBSA**
0.0	0.40	0.48	0.62
0.1	0.46	0.49	0.63
0.2	0.50	0.50	0.64
0.3	0.53	0.51	0.65
0.4	0.55	0.51	0.65
0.5	0.55	0.52	0.66
**0.6**	**0.54**	**0.53**	**0.67**
0.7	0.51	0.53	0.67
0.8	0.48	0.54	0.68
0.9	0.45	0.55	0.69
1.0	0.42	0.55	0.69

Table S6. Correlation coefficients (R^2) for the Test dataset from VC, MM-GBSA and MM-PBSA analyses post-MD or pre-MD with energy minimization only (with/without ligand conformational entropies).

Method	MD[a] Moiety RMSD < 2 Å	MD[b] Moiety RMSD 2–3 Å	MD[c] Moiety RMSD > 3 Å	MD All Poses	EMin GA	EMin X-tal
MM-PBSA	0.67/0.62	0.32/0.28	0.00/0.03	0.45/0.41	0.22/0.18	0.26/0.21
MM-GBSA	0.53/0.49	0.35/0.32	0.08/0.14	0.35/0.32	0.27/0.22	0.28/0.23
VC	0.54/0.40	0.37/0.23	0.12/0.11	0.42/0.26	0.18/0.08	0.17/0.08

Table S7. MM-GBSA[a] Energies (kcal/mol) for calcium–ligand interactions in calcium-containing lectins.

Receptor	PDB ID	E_{vdW}	E_{Ele}	$E_{GBSA\text{-}Pol}$	MM-GBSA
E-selectin	4C16	2.3 ± 0.9	-15.5 ± 0.9	19.3 ± 1.1	6.1 ± 1.3
	6EYI	2.5 ± 1.0	-16.2 ± 0.9	19.5 ± 1.0	5.8 ± 1.3
	6EYJ	2.2 ± 1.0	-14.7 ± 0.9	18.8 ± 1.1	6.3 ± 1.3
	8R5M	2.3 ± 0.9	-14.8 ± 0.9	18.7 ± 1.0	6.1 ± 1.3
DC-SIGN	6GHV	1.7 ± 0.8	4.1 ± 1.0[b]	1.7 ± 1.1	7.4 ± 1.3
	7NL6	2.0 ± 0.9	-5.4 ± 1.0	10.8 ± 1.1	7.4 ± 1.4
	7NL7	1.9 ± 0.9	4.8 ± 1.0[b]	1.0 ± 1.1	7.6 ± 1.3
	9EMR	2.1 ± 0.9	-6.1 ± 1.2	11.3 ± 1.2	7.4 ± 1.4
	9EMQ	1.8 ± 0.8	5.0 ± 1.0[b]	0.7 ± 1.1	7.5 ± 1.3
LecA	5MIH	3.2 ± 1.1	-8.3 ± 0.7	14.5 ± 1.0	9.4 ± 1.5
	7Z62	3.0 ± 1.1	-8.4 ± 0.7	14.4 ± 1.0	9.0 ± 1.5
	4LJH	3.0 ± 1.1	-8.8 ± 0.7	14.5 ± 1.0	8.7 ± 1.5
	4LK7	2.8 ± 1.1	-7.8 ± 0.7	13.6 ± 1.0	8.6 ± 1.5
	3ZYF	3.0 ± 1.1	-7.1 ± 0.7	12.9 ± 1.0	8.9 ± 1.5
	4A6S	3.0 ± 1.1	-8.2 ± 0.7	14.0 ± 1.0	8.8 ± 1.5
LecB	5MB1	3.6 ± 1.8	-15.6 ± 1.9	25.4 ± 2.5	13.4 ± 2.9
	5MAY	3.8 ± 1.8	-14.8 ± 1.9	25.2 ± 2.5	14.1 ± 3.0
	8AIJ	4.6 ± 2.0	-17.7 ± 1.9	28.8 ± 2.4	15.7 ± 2.9
	8AIY	4.3 ± 1.9	-16.8 ± 1.7	27.9 ± 2.3	15.3 ± 2.9
	5MAZ	3.8 ± 1.8	-15.1 ± 2.0	24.8 ± 2.5	13.5 ± 3.0
	3ZDV	4.5 ± 2.0	-17.1 ± 2.0	28.0 ± 2.5	15.3 ± 2.9
	5A3O	4.3 ± 1.9	-16.9 ± 1.7	27.5 ± 2.2	14.8 ± 2.9
**Average**		**3.0 ± 1.3**	**-10.1 ± 1.2**	**17.0 ± 1.5**	**9.9 ± 1.9**

[a]Non-polar GBSA values were all <0.1 kcal/mol and are not shown. [b]Ligand contains a positively-charged moiety.

Table S8. System information of the Application dataset.

Receptor	Protein-carbohydrate PDB ID	PDB Chain	Retained Crystallographic Water Number(s)	Experimental Affinity Method	Reference	Compound Name	Glycomimetic Identifier
DC-SIGN	2IT6	A	None	ITC	Cramer et al (4)	11	A1
						19	A2
						1l	A3
						1n	A4
						1o	A5
						1p	A6
						1q	A7
						1r	A8
						1s	A9
						21	A10
						23	A11
						25	A12
						9	A13
Galectin-1	3OYW	A	137, 138	FP	Delaine et al (21)	10	A14
						11	A15
						12	A16
						2	A17
						3	A18
						4	A19
						5	A20
						6	A21
						7	A22
						8	A23
						9	A24
	4JC1	A	251	FP		10	A25

Galectin-3					Delaine et al (21)	11	A26
						12	A27
						17	A28
						2	A29
						3	A30
						4	A31
						5	A32
						6	A33
						7	A34
						8	A35
						9	A36
LecB	1OUR	A	484, 485, 486, 495	FP	Hauck et al (27)	2a	A37
						2b	A38
						2c	A39
						2d	A40
						2e	A41
						2g	A42
						3a	A43
						3b	A44
						3c	A45
						3d	A46
						4a	A47
						4b	A48
						4c	A49
						4d	A50
						4e	A51
						4f	A52
						4g	A53
						5a	A54
						5b	A55

						5c	A56
						5d	A57
						5e	A58
						5g	A59
LecA	4A6S	A	2054, 2081	ITC	Kadam et al (35)	10	A60
						11	A61
						12	A62
						13	A63
						14	A64
						15	A65
						16	A66
						17	A67
						2	A68
						4	A69
						5	A70
						6	A71
						8	A72
						9	A73

Table S9. Calculated ligand binding energies (kcal/mol) for the Application dataset computed with VC, MM-GBSA, and MM-PBSA not including entropy corrections.

Glycomimetic Identifier	Pre-MD Interaction Energy			Post-MD Interaction Energy			Experimental
	VC	MM-GBSA	MM-PBSA	VC	MM-GBSA	MM-PBSA	
A1	-5.9	-20.7	-13.2	-5.4	-9.9	-0.3	-5.1
A2	-6.9	-33.1	-24.8	-5.8	-11.4	-2.0	-6.2
A3	-6.3	-24.1	-19.2	-5.2	-10.2	-1.2	-4.2
A4	-6.9	-19.6	-14.0	-5.3	-9.7	0.4	-4.5
A5	-7.5	-30.4	-21.6	-5.1	-8.1	2.6	-4.9
A6	-6.6	-22.1	-14.2	-5.4	-8.9	3.0	-4.8
A7	-7.6	-29.0	-17.7	-5.8	-11.3	0.8	-4.7
A8	-5.8	-15.5	-9.7	-5.2	-9.3	1.5	-4.9
A9	-5.8	-13.3	-4.7	-5.1	-7.8	1.8	-5.2
A10	-7.5	-32.2	-15.5	-5.9	-10.0	2.1	-6.0
A11	-7.2	-28.6	-18.2	-6.3	-15.8	-8.3	-5.3
A12	-8.2	-29.4	-12.3	-6.2	-11.1	2.9	-4.7
A13	-6.8	-32.0	-21.0	-5.8	-10.5	1.0	-6.1
A14	-7.5	-57.9	-22.3	-13.7	-57.1	-18.6	-5.4
A15	-8.3	-67.9	-28.7	-10.6	-41.2	-6.0	-5.6
A16	-3.5	-44.2	-12.1	-11.9	-56.6	-28.6	-10.9
A17	-5.6	-58.6	-23.0	-12.5	-56.1	-28.3	-10.0
A18	-8.8	-57.9	-28.9	-9.8	-38.8	-10.1	-8.9
A19	-5.4	-49.7	-20.3	-12.7	-53.7	-25.4	-10.8
A20	-6.7	-56.6	-25.1	-12.8	-54.7	-24.2	-10.3
A21	-5.6	-59.0	-26.9	-8.8	-37.5	-7.5	-8.8
A22	-6.4	-57.3	-25.3	-12.6	-52.9	-22.7	-8.0
A23	-8.3	-54.8	-21.5	-14.5	-57.0	-28.9	-8.7
A24	-6.9	-54.2	-22.5	-13.3	-52.3	-23.7	-8.1
A25	-14.0	-60.4	-10.7	-12.7	-47.5	-22.8	-4.2
A26	-14.0	-55.6	-24.0	-12.2	-41.6	-16.8	-8.8
A27	-11.6	-46.4	-21.2	-10.5	-39.7	-16.5	-9.8
A28	-14.4	-58.6	-31.0	-11.8	-41.8	-14.5	-8.2
A29	-12.6	-49.3	-24.9	-9.5	-35.3	-15.9	-10.0
A30	-12.5	-50.3	-22.2	-9.9	-40.2	-18.8	-9.2
A31	-13.5	-53.9	-26.5	-10.0	-40.1	-20.0	-10.7
A32	-12.8	-35.0	-9.2	-10.7	-45.8	-25.1	-10.2
A33	-13.4	-50.0	-24.1	-11.6	-41.0	-15.0	-9.2
A34	-13.3	-55.6	-26.9	-11.3	-42.8	-20.9	-8.8
A35	-13.7	-50.0	-25.9	-11.8	-39.1	-17.5	-9.1
A36	-10.8	-41.7	-15.4	-10.8	-37.2	-16.7	-9.0
A37	-3.4	-2.2	2.4	-5.4	5.4	9.4	-4.4
A38	-4.0	-7.3	4.2	-5.8	4.0	7.1	-4.9
A39	-3.6	-6.5	7.0	-5.6	4.2	7.9	-4.7

A40	-3.8	-7.1	2.4	-5.8	3.3	7.2	-4.8
A41	-3.4	-7.5	-0.1	-6.0	1.1	8.7	-4.8
A42	-3.5	-8.6	1.6	-5.8	5.0	8.1	-4.9
A43	-4.1	-10.3	1.7	-5.6	3.8	12.1	-5.0
A44	-4.6	-13.0	-0.2	-6.0	4.4	6.4	-5.4
A45	-3.8	-6.7	5.1	-6.1	2.2	6.4	-5.4
A46	-4.0	-9.8	5.1	-5.6	4.5	8.0	-5.0
A47	-2.3	-0.7	6.8	-5.2	6.4	8.0	-4.8
A48	-3.6	-3.0	6.9	-5.4	5.6	9.9	-5.4
A49	-3.4	-3.3	5.5	-6.6	2.5	10.8	-5.6
A50	-4.4	-5.0	3.1	-5.8	4.5	7.2	-5.3
A51	-4.5	-6.9	2.5	-6.0	6.0	11.4	-5.6
A52	-4.2	-4.0	12.7	-6.5	6.2	9.2	-5.2
A53	-3.9	-4.7	4.4	-6.7	2.3	4.7	-6.0
A54	-5.0	-4.3	1.1	-6.1	5.7	11.7	-6.5
A55	-3.5	-8.2	4.4	-6.1	3.2	7.4	-5.9
A56	-4.8	-10.3	3.7	-5.9	4.4	7.6	-6.4
A57	-5.7	-9.0	1.7	-6.1	6.0	9.4	-6.1
A58	-4.9	-11.1	4.6	-6.7	1.5	9.6	-7.5
A59	-5.3	-8.8	7.8	-6.9	2.2	7.0	-5.5
A60	-5.2	-11.1	-1.8	-6.3	-7.2	0.9	-7.0
A61	-4.0	-9.9	-2.7	-6.0	-8.0	-0.2	-6.8
A62	-6.0	-13.1	-2.7	-6.4	-7.0	3.0	-7.3
A63	-5.2	-12.4	-3.6	-6.8	-7.4	1.3	-7.3
A64	-5.5	-15.3	-5.0	-6.6	-8.1	1.3	-7.2
A65	-3.7	-9.9	5.7	-6.6	-9.5	0.4	-6.4
A66	-4.8	-16.3	-4.8	-6.8	-7.1	2.4	-6.9
A67	-2.8	-14.4	-4.8	-7.2	-9.2	2.4	-7.1
A68	-3.0	-9.0	-2.6	-5.9	-6.9	0.0	-6.1
A69	-5.7	-10.7	5.5	-6.8	-6.0	4.9	-5.9
A70	-5.3	-11.3	-0.4	-6.1	-7.1	0.4	-6.6
A71	-3.8	-13.0	-5.3	-6.3	-6.7	1.4	-6.6
A72	-3.2	-11.3	-6.8	-6.2	-7.8	-0.1	-6.9
A73	-3.4	-10.8	-3.7	-6.5	-9.0	-0.3	-6.9

Table S10. Calculated ligand binding energies (kcal/mol) from Table S9 including conformational entropies (0.6 kcal/mol per rotatable bond).

Identifier	N_{rot} Moiety	$-T\Delta S_{lig}$	Pre-MD			Post-MD			Experimental
			VC	MM-GBSA	MM-PBSA	VC	MM-GBSA	MM-PBSA	
A1	0	0.0	-5.9	-20.7	-13.2	-5.4	-9.9	-0.3	-5.1
A2	2	1.2	-5.7	-31.9	-23.6	-4.6	-10.2	-0.8	-6.2
A3	1	0.6	-5.7	-23.5	-18.6	-4.6	-9.6	-0.6	-4.2
A4	5	3.0	-3.9	-16.6	-11.0	-2.3	-6.7	3.4	-4.5
A5	5	3.0	-4.5	-27.4	-18.6	-2.1	-5.1	5.6	-4.9
A6	3	1.8	-4.8	-20.3	-12.4	-3.6	-7.1	4.8	-4.8
A7	2	1.2	-6.4	-27.8	-16.5	-4.6	-10.1	2.0	-4.7
A8	0	0.0	-5.8	-15.5	-9.7	-5.2	-9.3	1.5	-4.9
A9	0	0.0	-5.8	-13.3	-4.7	-5.1	-7.8	1.8	-5.2
A10	1	0.6	-6.9	-31.6	-14.9	-5.3	-9.4	2.7	-6.0
A11	1	0.6	-6.6	-28.0	-17.6	-5.7	-15.2	-7.7	-5.3
A12	1	0.6	-7.6	-28.8	-11.7	-5.6	-10.5	3.5	-4.7
A13	2	1.2	-5.6	-30.8	-19.8	-4.6	-9.3	2.2	-6.1
A14	0	0.0	-7.5	-57.9	-22.3	-13.7	-57.1	-18.6	-5.4
A15	4	2.4	-5.9	-65.5	-26.3	-8.2	-38.8	-3.6	-5.6
A16	0	0.0	-3.5	-44.2	-12.1	-11.9	-56.6	-28.6	-10.9
A17	0	0.0	-5.6	-58.6	-23.0	-12.5	-56.1	-28.3	-10.0
A18	0	0.0	-8.8	-57.9	-28.9	-9.8	-38.8	-10.1	-8.9
A19	0	0.0	-5.4	-49.7	-20.3	-12.7	-53.7	-25.4	-10.8
A20	0	0.0	-6.7	-56.6	-25.1	-12.8	-54.7	-24.2	-10.3
A21	0	0.0	-5.6	-59.0	-26.9	-8.8	-37.5	-7.5	-8.8
A22	0	0.0	-6.4	-57.3	-25.3	-12.6	-52.9	-22.7	-8.0
A23	0	0.0	-8.3	-54.8	-21.5	-14.5	-57.0	-28.9	-8.7
A24	0	0.0	-6.9	-54.2	-22.5	-13.3	-52.3	-23.7	-8.1

A25	0	0.0	-14.0	-60.4	-10.7	-12.7	-47.5	-22.8	-4.2
A26	4	2.4	-11.6	-53.2	-21.6	-9.8	-39.2	-14.4	-8.8
A27	0	0.0	-11.6	-46.4	-21.2	-10.5	-39.7	-16.5	-9.8
A28	0	0.0	-14.4	-58.6	-31.0	-11.8	-41.8	-14.5	-8.2
A29	0	0.0	-12.6	-49.3	-24.9	-9.5	-35.3	-15.9	-10.0
A30	0	0.0	-12.5	-50.3	-22.2	-9.9	-40.2	-18.8	-9.2
A31	0	0.0	-13.5	-53.9	-26.5	-10.0	-40.1	-20.0	-10.7
A32	0	0.0	-12.8	-35.0	-9.2	-10.7	-45.8	-25.1	-10.2
A33	0	0.0	-13.4	-50.0	-24.1	-11.6	-41.0	-15.0	-9.2
A34	0	0.0	-13.3	-55.6	-26.9	-11.3	-42.8	-20.9	-8.8
A35	0	0.0	-13.7	-50.0	-25.9	-11.8	-39.1	-17.5	-9.1
A36	0	0.0	-10.8	-41.7	-15.4	-10.8	-37.2	-16.7	-9.0
A37	1	0.6	-2.8	-1.6	3.0	-4.8	6.0	10.0	-4.4
A38	3	1.8	-2.2	-5.5	6.0	-4.0	5.8	8.9	-4.9
A39	1	0.6	-3.0	-5.9	7.6	-5.0	4.8	8.5	-4.7
A40	1	0.6	-3.2	-6.5	3.0	-5.2	3.9	7.8	-4.8
A41	1	0.6	-2.8	-6.9	0.5	-5.4	1.7	9.3	-4.8
A42	1	0.6	-2.9	-8.0	2.2	-5.2	5.6	8.7	-4.9
A43	2	1.2	-2.9	-9.1	2.9	-4.4	5.0	13.3	-5.0
A44	2	1.2	-3.4	-11.8	1.0	-4.8	5.6	7.6	-5.4
A45	2	1.2	-2.6	-5.5	6.3	-4.9	3.4	5.2	-5.4
A46	2	1.2	-2.8	-8.6	6.3	-4.4	5.7	9.2	-5.0
A47	0	0.0	-2.3	-0.7	6.8	-5.2	6.4	8.0	-4.8
A48	0	0.0	-3.6	-3.0	6.9	-5.4	5.6	9.9	-5.4
A49	0	0.0	-3.4	-3.3	5.5	-6.6	2.5	10.8	-5.6
A50	0	0.0	-4.4	-5.0	3.1	-5.8	4.5	7.2	-5.3
A51	2	1.2	-3.3	-5.7	3.7	-4.8	7.2	12.6	-5.6
A52	3	1.8	-3.6	-2.2	14.5	-4.7	8.0	11.0	-5.2
A53	0	0.0	-3.9	-4.7	4.4	-6.7	2.3	4.7	-6.0
A54	2	1.2	-3.8	-3.1	2.3	-4.9	6.9	12.9	-6.5
A55	2	1.2	-2.3	-7.0	5.6	-4.9	4.4	8.6	-5.9

A56	2	1.2	-3.6	-9.1	4.9	-4.7	5.6	8.8	-6.4
A57	2	1.2	-4.5	-7.8	2.9	-4.9	7.2	10.6	-6.1
A58	2	1.2	-3.7	-9.9	5.8	-5.5	2.7	10.8	-7.5
A59	2	1.2	-4.1	-7.6	9.0	-5.7	3.4	8.2	-5.5
A60	1	0.6	-4.6	-10.5	-1.2	-5.7	-6.6	1.5	-7.0
A61	1	0.6	-3.4	-9.3	-2.1	-5.4	-7.4	0.4	-6.8
A62	1	0.6	-5.4	-12.5	-2.1	-5.8	-6.4	3.6	-7.3
A63	1	0.6	-4.6	-11.8	-3.0	-6.2	-6.8	1.9	-7.3
A64	1	0.6	-4.9	-14.7	-4.4	-6.0	-7.5	1.9	-7.2
A65	1	0.6	-3.1	-9.3	6.3	-6.0	-8.9	1.0	-6.4
A66	1	0.6	-4.2	-15.7	-4.2	-6.2	-6.5	3.0	-6.9
A67	4	2.4	-0.4	-12.0	-2.4	-4.8	-6.8	4.8	-7.1
A68	1	0.6	-2.4	-8.4	-2.0	-5.3	-6.3	0.6	-6.1
A69	3	1.8	-3.9	-8.9	7.3	-5.0	-4.2	6.7	-5.9
A70	3	1.8	-3.5	-9.5	1.4	-4.3	-5.3	2.2	-6.6
A71	1	0.6	-3.2	-12.4	-4.7	-5.7	-6.1	2.0	-6.6
A72	1	0.6	-2.6	-10.7	-6.2	-5.6	-7.2	0.5	-6.9
A73	1	0.6	-2.8	-10.2	-3.1	-5.9	-8.4	0.3	-6.9

Guidance For Technical Readers

The execution of GA begins with the *MonteCarlo*() function located in *Internal/gm/monte_carlo.hpp*. The grafting method is executed with the *GraftMoietyAndRemoveDummyAtoms()* function in *Internal/gm/utility.hpp.* The pdb2glycam workflow is invoked with a call of the *pdb2glycam_matching()* function, which is located in *Internal/gm/pdb2glycam.hpp*. This function uses other GMML helper functions extensively, especially the *MatchPdbAtoms2Glycam()* function, where subgraph isomorphism matching occurs. The entry point of the GM2MD workflow is located in *Internal/gm2md/main.cpp*, which calls the submodules described in Figure S2 sequentially. The source code of the ScoreTraj program resides in *Internal/scoretraj/scoretraj.cpp*. PDBQT files of moieties in the virtual library are stored at *Data/moieties/pdbqt/Yao/virtual_screening* and *Data/moieties/pdbqt/Alex*, with the names of the subdirectories indicating the identity of the functional group.

References

1. E. Fan *et al.*, Exploration of the GM1 receptor-binding site of heat-labile enterotoxin and cholera toxin by phenyl-ring-containing galactose derivatives. *Acta Crystallogr. D Biol. Crystallogr.* **57**, 201-212 (2001).
2. D. D. Mitchell, J. C. Pickens, K. Korotkov, E. Fan, W. G. Hol, 3, 5-Substituted phenyl galactosides as leads in designing effective cholera toxin antagonists: synthesis and crystallographic studies. *Bioorg. Med. Chem.* **12**, 907-920 (2004).
3. J. C. Pickens *et al.*, Nonspanning bivalent ligands as improved surface receptor binding inhibitors of the cholera toxin B pentamer. *Cell Chem. Biol.* **11**, 1205-1215 (2004).
4. J. Cramer *et al.*, Sweet drugs for bad bugs: a glycomimetic strategy against the DC-SIGN-mediated dissemination of SARS-CoV-2. *J. Am. Chem. Soc.* **143**, 17465-17478 (2021).

5. L. Medve *et al.*, Enhancing Potency and Selectivity of a DC-SIGN Glycomimetic Ligand by Fragment-Based Design: Structural Basis. *Chem. Eur. J.* **25**, 14659-14668 (2019).
6. D. D. Nemli *et al.*, Thermodynamics-Guided Design Reveals a Cooperative Hydrogen Bond in DC-SIGN-targeted Glycomimetics. *J. Med. Chem.* **67**, 13813-13828 (2024).
7. S. Kleeb *et al.*, FimH antagonists: bioisosteres to improve the in vitro and in vivo PK/PD profile. *J. Med. Chem.* 2015, 58, 5, 2221–2239 (2015).
8. S. Vanwetswinkel *et al.*, Study of the structural and dynamic effects in the FimH adhesin upon α-d-heptyl mannose binding. *J. Med. Chem.* **57**, 1416-1427 (2014).
9. B. Fiege *et al.*, The tyrosine gate of the bacterial lectin FimH: a conformational analysis by NMR spectroscopy and X-ray crystallography. *ChemBioChem* **16**, 1235-1246 (2015).
10. J. Bouckaert *et al.*, Receptor binding studies disclose a novel class of high-affinity inhibitors of the Escherichia coli FimH adhesin. *Mol. Microbiol.* **55**, 441-455 (2005).
11. V. Kalas *et al.*, Structure-based discovery of glycomimetic FmlH ligands as inhibitors of bacterial adhesion during urinary tract infection. *Proc. Natl. Acad. Sci. U. S. A.* **115**, E2819-E2828 (2018).
12. T.-J. Hsieh *et al.*, Dual thio-digalactoside-binding modes of human galectins as the structural basis for the design of potent and selective inhibitors. *Sci. Rep.* **6**, 29457 (2016).
13. K. Peterson *et al.*, Aromatic heterocycle galectin-1 interactions for selective single-digit nM affinity ligands. *RSC Adv.* **8**, 24913-24922 (2018).
14. F. R. Zetterberg *et al.*, Discovery of the selective and orally available galectin-1 inhibitor GB1908 as a potential treatment for lung cancer. *J. Med. Chem.* (2024).
15. N. Bertleff-Zieschang *et al.*, Exploring the Structural Space of the Galectin-1–Ligand Interaction. *ChemBioChem* **18**, 1477-1481 (2017).

16. P. Sörme *et al.*, Structural and thermodynamic studies on cation− π interactions in lectin−ligand complexes: high-affinity galectin-3 inhibitors through fine-tuning of an arginine−arene interaction. *J. Am. Chem. Soc.* **127**, 1737-1743 (2005).
17. P. M. Collins, C. T. Öberg, H. Leffler, U. J. Nilsson, H. Blanchard, Taloside Inhibitors of Galectin-1 and Galectin-3. *Chem. Biol. Drug Des.* **79**, 339-346 (2012).
18. V. K. Rajput *et al.*, A selective galactose–coumarin-derived galectin-3 inhibitor demonstrates involvement of galectin-3-glycan interactions in a pulmonary fibrosis model. *J. Med. Chem.* **59**, 8141-8147 (2016).
19. C. Atmanene *et al.*, Biophysical and structural characterization of mono/di-arylated lactosamine derivatives interaction with human galectin-3. *Biochem. Biophys. Res. Commun.* **489**, 281-286 (2017).
20. C. Liu *et al.*, Identification of benzothiazole derived monosaccharides as potent, selective, and orally bioavailable inhibitors of human and mouse galectin-3; a rare example of using a S··· O binding interaction for drug design. *Bioorg. Med. Chem.* **101**, 117638 (2024).
21. T. Delaine *et al.*, Galectin-3-binding glycomimetics that strongly reduce bleomycin-induced lung fibrosis and modulate intracellular glycan recognition. *ChemBioChem* **17**, 1759-1770 (2016).
22. A. Kumar *et al.*, Molecular mechanism of interspecies differences in the binding affinity of TD139 to Galectin-3. *Glycobiology* **31**, 1390-1400 (2021).
23. R. U. Kadam *et al.*, A glycopeptide dendrimer inhibitor of the galactose-specific lectin LecA and of Pseudomonas aeruginosa biofilms. *Angew. Chem., Int. Ed. Engl.* **50**, 10631 (2011).

24. J. Rodrigue *et al.*, Aromatic thioglycoside inhibitors against the virulence factor LecA from Pseudomonas aeruginosa. *Org. Biomol. Chem.* **11**, 6906-6918 (2013).
25. S. Wagner *et al.*, Covalent lectin inhibition and application in bacterial biofilm imaging. *Angew. Chem. Int. Ed.* **56**, 16559-16564 (2017).
26. A. Bruneau *et al.*, Discovery of potent 1, 1-diarylthiogalactoside glycomimetic inhibitors of Pseudomonas aeruginosa LecA with antibiofilm properties. *Eur. J. Med. Chem.* **247**, 115025 (2023).
27. D. Hauck *et al.*, Discovery of two classes of potent glycomimetic inhibitors of Pseudomonas aeruginosa LecB with distinct binding modes. *ACS Chem. Biol.* **8**, 1775-1784 (2013).
28. R. Sommer *et al.*, Cinnamide Derivatives of d-Mannose as Inhibitors of the Bacterial Virulence Factor LecB from Pseudomonas aeruginosa. *ChemistryOpen* **4**, 756-767 (2015).
29. R. Sommer *et al.*, Glycomimetic, orally bioavailable LecB inhibitors block biofilm formation of Pseudomonas aeruginosa. *J. Am. Chem. Soc.* **140**, 2537-2545 (2018).
30. P. Mała *et al.*, Discovery of N-Β-L-fucosyl amides as high-affinity ligands for the Pseudomonas Aeruginosa lectin Lecb. *J. Med. Chem.* **65**, 14180-14200 (2022).
31. R. Sommer *et al.*, Anti-biofilm agents against Pseudomonas aeruginosa: a structure–activity relationship study of C-glycosidic LecB inhibitors. *J. Med. Chem.* **62**, 9201-9216 (2019).
32. H. Attrill *et al.*, The structure of siglec-7 in complex with sialosides: leads for rational structure-based inhibitor design. *Biochem. J.* **397**, 271-278 (2006).
33. N. Varga *et al.*, Strengthening an Intramolecular Non-Classical Hydrogen Bond to Get in Shape for Binding. *Angew. Chem. Int. Ed.* **63**, e202406024 (2024).

34. B. Wagner *et al.*, Analogues of the pan-selectin antagonist rivipansel (GMI-1070). *Eur. J. Med. Chem.* **272**, 116455 (2024).

35. R. U. Kadam *et al.*, CH− π "T-shape" interaction with Histidine explains binding of aromatic Galactosides to Pseudomonas aeruginosa Lectin LecA. *ACS Chem. Biol.* **8**, 1925-1930 (2013).
